# Comprehensive Review: Optimization of Epoxy Composites, Mechanical Properties, & Technological Trends

**DOI:** 10.3390/polym17030271

**Published:** 2025-01-22

**Authors:** Jozef Jaroslav Fekiač, Michal Krbata, Marcel Kohutiar, Róbert Janík, Lucia Kakošová, Alena Breznická, Maroš Eckert, Pavol Mikuš

**Affiliations:** 1 Faculty of Special Technology, Alexander Dubcek University of Trenčín, Ku Kyselke 469, 911 06 Trenčín, Slovakia; jozef.fekiac@tnuni.sk (J.J.F.); michal.krbata@tnuni.sk (M.K.); lucia.kakosova@tnuni.sk (L.K.); alena.breznicka@tnuni.sk (A.B.); maros.eckert@tnuni.sk (M.E.); pavol.mikus@tnuni.sk (P.M.); 2 Faculty of Industrial Technologies in Púchov, Alexander Dubček University of Trenčín, Ivana Krasku 491/30, 020 01 Púchov, Slovakia; robert.janik@tnuni.sk

**Keywords:** epoxy composites, mechanical properties, thermal stability, nanomaterials, hybrid reinforcement

## Abstract

Epoxy composites play a crucial role in modern materials technologies, with their exceptional properties such as high strength and thermal and chemical resistance, making them ideal for a wide range of industrial applications, including aerospace, automotive, construction, and energy. This review article provides a comprehensive overview of the current trends and advancements in epoxy composites, focusing on mechanical properties and their optimization. Attention is given to technological innovations, including the use of nanotechnologies, hybrid reinforcement, and eco-friendly materials, which are key to enhancing the performance and sustainability of these materials. The analysis shows that the introduction of nanomaterials, such as graphene, titanium dioxide, and silicon dioxide, can significantly improve the strength, fatigue resistance, and electrical properties of epoxy composites, opening new possibilities in advanced technologies. Another significant contribution is the development of hybrid composites, which combine different types of fibers, such as carbon, aramid, and glass fibers, enabling the optimization of key properties, including interlayer strength and delamination resistance. The article also highlights the importance of environmental innovations, such as bio-based resins and self-healing mechanisms, which enable more sustainable and long-term effective use of composites. The combination of theoretical knowledge with practical applications provides valuable guidance for designing materials with precisely defined properties for future industrial use. This text thus offers a comprehensive view of the possibilities of epoxy composites in the context of increasing demands for performance, reliability, and environmental sustainability.

## 1. Introduction

Epoxy composites represent a key element of modern material technologies, playing a significant role across various industrial sectors. Due to their exceptional properties, such as high strength, thermal resistance, and chemical stability, they are widely used in the aerospace and automotive industries, construction, energy production, and other fields. The aim of this text is to analyze the mechanical properties of epoxy composites and explore optimization methods that enhance their performance and sustainability in applications with demanding requirements [1,2,3,4,5].

A crucial aspect of epoxy composite research lies in their ability to interact with various types of fillers and fibrous materials, enabling the achievement of specific properties tailored to application needs. For instance, the incorporation of nanoparticles such as titanium dioxide (TiO_2_) or silicon dioxide (SiO_2_) significantly improves strength, fatigue resistance, and other mechanical parameters. Furthermore, the integration of graphene or graphene oxide (GO) into epoxy resin matrices leads to a notable increase in electrical conductivity and resistance to thermal shocks, opening new possibilities in electronics and other advanced technologies [2,5,6,7,8].

Another important direction is the development of hybrid composites that combine different types of fibers, such as carbon, aramid, and glass fibers. This combination allows for the optimization of properties such as interlayer strength, stiffness, and delamination resistance. Modern technologies also enable surface treatments of fibers to enhance their adhesion to epoxy resins, minimizing the risk of microcracks under stress. These innovations significantly contribute to improving the reliability and durability of epoxy composites in practice [3,4,7,9].

The environmental aspect is an increasingly important part of epoxy composite development. Research on bio-based resins, derived from sources like lignin or soybean oil, offers a sustainable alternative to traditional petroleum-based derivatives. These resins retain their mechanical properties and present new opportunities for eco-friendly applications. Similarly, self-healing mechanisms are of interest, extending the lifespan of composites by enabling the repair of microcracks and structural defects during use [5,6,9,10].

Another critical factor is the tribological behavior of epoxy composites. High wear resistance, achieved through the addition of nanoparticles, is essential for applications involving intense friction, such as bearings and gears. The control of curing processes, which affects the final mechanical and thermal properties, represents another step toward optimizing composites for specific uses [1,4,7,11].

The objective of this article is to provide a comprehensive overview of the current state and developmental trends in epoxy composites. This text focuses on a detailed analysis of the mechanical properties of epoxy composites and the identification of innovative solutions that could significantly enhance their performance in a wide range of industrial applications. Special attention is given to technological innovations such as nanotechnologies, hybrid reinforcement, and environmentally friendly materials, which offer the potential for sustainable and efficient utilization of these composites. Additionally, this text provides a thorough review of the latest approaches to optimizing the properties of epoxy composites, contributing to their effective application in demanding conditions. Emphasis is placed on linking theoretical knowledge with practical applications, enabling the design of materials with precisely defined properties. This approach also opens new perspectives for expanding their usability in the future, reinforcing their importance in modern materials science [2,5,6,7,9,12].

## 2. Mechanical Properties of Epoxy Composites and Their Optimization: Enhancing the Strength Properties of Epoxy Polymers

Epoxy composites are fundamental materials for industrial applications due to their high strength and durability. Their mechanical properties are enhanced by the addition of nanomaterials, such as graphene oxide, which increases tensile strength and modulus of elasticity. Short-fiber and hybrid graphene composites combine fibrous and particulate fillers, achieving a synergistic effect that improves resistance to delamination and cracking. Hybrid reinforcement, such as the use of polyaryletherketone interlayers, further enhances toughness and ensures better stress transfer within the material. These innovations enable the development of composites with outstanding properties for demanding conditions [1,2,13,14].

Epoxy polymers are among the essential materials used in modern industrial applications, such as aerospace, construction, and the automotive industry, owing to their high strength, resistance to chemical corrosion, and stability under load [1,2,3,5]. Improving their properties is a subject of intensive research, with significant attention devoted to the addition of particulate and fibrous fillers, the development of environmentally friendly hardeners, and the utilization of nanotechnologies. Notable advancements have been made, for instance, with the use of graphene oxide (GO) and the combination of conventional fillers with nanomaterials such as nano-TiO_2_ [13,14]. Research by Kohutiar et al. confirms that the development of bio-based hardeners is equally crucial, offering ecological solutions without compromising mechanical properties [15].

**Figure 1:**
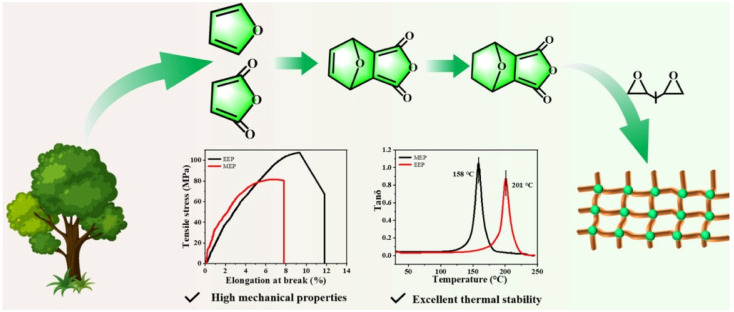
The Production Process of Bio-Composites: Transforming Natural Raw Materials into a Polymer Network with Enhanced Mechanical Properties and Excellent Thermal Stability [15].

Epoxy resins, based on thermosetting polymers, are widely used in coatings, electronic components, adhesives, and composite materials in the automotive and aerospace industries. A critical step in their production is the selection of suitable hardeners to ensure the desired mechanical and thermal properties. Commercial anhydride hardeners are frequently used due to their low toxicity and high reactivity; however, they are predominantly petroleum-derived, leading to significant CO_2_ emissions [16]. New research, therefore, focuses on bio-based hardeners that could replace traditional petroleum-based products. An example is the novel bio-based anhydride (EHPA), which exhibits excellent tensile strength (105.51 MPa), modulus of elasticity (2.33 GPa), and thermal stability (Td5 = 329 °C). Additionally, its structure contains ester bonds, allowing easy degradability, thereby supporting the ecological use of materials in the aerospace and defense industries [15]. Figure 1 illustrates the process of bio-composite production, demonstrating the transformation of natural raw materials into a polymer network. This process results in materials with enhanced mechanical properties and excellent thermal stability.

Another significant advancement is the utilization of graphene oxide (GO) to enhance the mechanical properties of epoxy composites. The addition of 1.5 vol.% GO improves tensile strength, hardness, and Young’s modulus, while higher GO contents (up to 6 vol.%) increase the homogeneity of the composite [13]. Research by Bartosova et al. demonstrated that GO can significantly enhance the properties of epoxy resins without the need for solvents, simplifying the production process and reducing its environmental impact [14].

In addition to the development of bio-based hardeners and nanomaterials, the optimization of fiber and particulate filler combinations is also crucial. Fibrous fillers, such as carbon fibers, provide high tensile strength, while particulate fillers, such as silicon dioxide nanoparticles, enhance compressive strength and improve flexural resistance. This synergistic effect results in the creation of composites capable of withstanding various types of mechanical loading [1,3].

**Figure 2:**
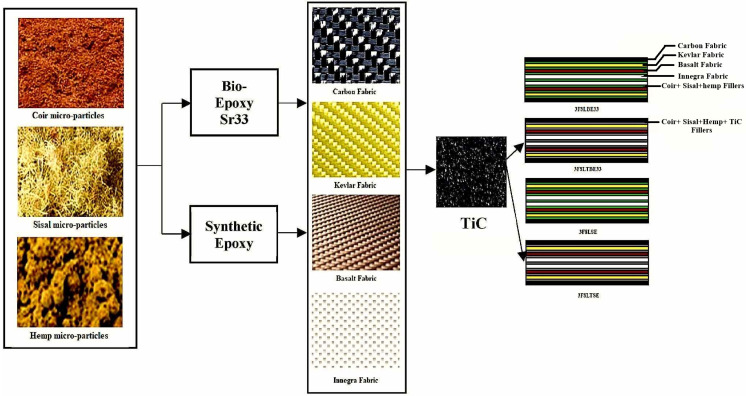
Schematic representation of the production of a bio/synthetic epoxy hybrid composite [12].

Figure 2 illustrates the schematic process of producing bio/synthetic epoxy hybrid composites, which integrates different types of fibrous and particulate fillers to achieve optimal properties. Research by Su et al. shows that the addition of silicon dioxide nanoparticles improves the load distribution within the epoxy matrix, reducing the risk of microcracks and increasing the material’s lifespan [2].

The combination of conventional fillers, such as PTFE, graphite, and carbon fibers, with nanomaterials like nano-TiO_2_ offers additional possibilities for improving the wear resistance of epoxy composites. An optimized composition (e.g., 15 vol.% graphite, 5 vol.% nano-TiO_2_, and 15 vol.% short carbon fibers) reduced the wear rate by up to 100 times compared to a pure epoxy matrix [14]. Such nanocomposites exhibit unique wear mechanisms, including a nanoscale “rolling effect” that protects the surface from severe damage. These results suggest significant potential for industrial applications [13,14].

In conclusion, the integration of advanced fillers, such as graphene oxide [13,14], with bio-based hardeners [16] and the synergistic use of fibrous and particulate fillers [1,2,3] represent an innovative solution for developing epoxy polymers with high strength, thermal stability, and ecological benefits. These materials hold considerable potential for structural applications where the combination of strength, durability, and sustainability is critical [1,2,3,12,13,14,15].

### 2.1. Mechanical Properties of Short-Fiber and Carbon-Fiber Composites

The mechanical properties of composites are one of the primary research focuses in modern materials science. Short-fiber polymer composites and carbon-fiber composites offer unique characteristics that make them highly attractive for applications in aerospace, automotive, and energy sectors. Their combination of low weight, high strength, and specific functional properties creates new possibilities for the development of advanced materials [17,18].

Additive manufacturing (AM) represents a cutting-edge technology that enables the layer-by-layer assembly of materials to create objects based on 3D models. This process allows for the production of prototypes and functional components with complex geometries that would be challenging or even impossible to fabricate using traditional methods. AM reduces production cycles, lowers costs, and enhances competitiveness, with its most prominent applications in the aerospace, automotive, and medical industries [19,20]. One of the most widely used AM technologies is fused filament fabrication (FFF), which involves the layer-by-layer deposition of thermoplastic materials such as ABS, PC, PLA, or PA. To improve the mechanical properties of these thermoplastics, carbon fibers are incorporated, resulting in carbon-fiber-reinforced plastic (CFRP) composites. These fibers enhance the strength and stiffness of the material, while the thermoplastic matrix binds them together and transfers applied loads [19].

Short-fiber polymer composites provide an excellent performance-to-weight ratio, although their properties are heavily influenced by microstructural factors such as fiber orientation, fiber length, and reinforcement volume fraction. Advanced numerical simulations based on Gusev’s approach and Monte Carlo algorithms enable precise modeling of these parameters, leading to improved predictions of elastic and thermoelastic properties. These methods have demonstrated that the average fiber length can effectively represent its distribution without significantly affecting the results [21,22]. Mathematical models, such as Halpin–Tsai or Mori–Tanaka, play a crucial role in predicting the properties of short-fiber composites. The Mori–Tanaka model has proven reliable for higher aspect ratios of fibers or greater volume fractions, facilitating efficient material designs with optimized properties [23,24].

**Figure 3:**
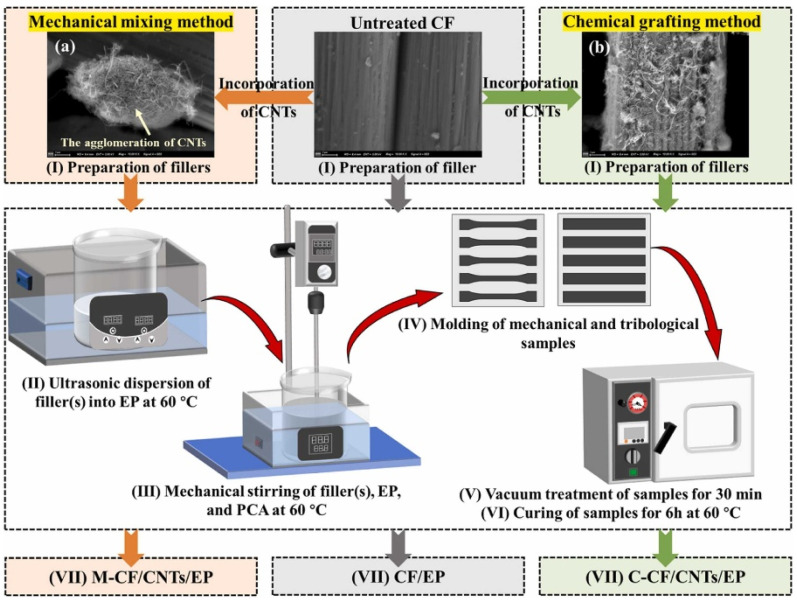
The manufacturing process of CF/CNTs/EP composites: (**a**) mechanical mixing of CNTs; (**b**) chemical grafting of CNTs; (**I**) filler preparation; (**II**) ultrasonic dispersion; (**III**) mechanical mixing; (**IV**) sample molding; (**V**) vacuum processing; (**VI**) curing [25].

Carbon composites, as demonstrated by the research of Guo et al., offer an additional dimension of functional properties, particularly in applications with high mechanical and tribological demands. Epoxy resins reinforced with carbon fibers and carbon nanotubes (CNTs) exhibit significantly improved tribological properties. CNTs reduce the coefficient of friction (COF) and wear rate (WR) by forming a uniform lubricating film, which extends the material’s lifespan and enhances its resistance to deformation [18]. Chemical grafting of CNTs onto the surface of carbon fibers promotes better dispersion and stronger bonding between the fibers and the matrix, thereby improving the composite’s mechanical stability [18,20]. Figure 3 illustrates the production process of CF/CNTs/EP composites, which includes mechanical mixing of CNTs (a), chemical grafting of CNTs (b), ultrasonic dispersion, mechanical mixing, sample molding, vacuum processing, and curing. These processes are critical to ensuring a uniform structure and optimized properties of the composite [25].

The addition of carbon fibers to thermoplastics in FFF composites has demonstrated significant increases in tensile strength and modulus of elasticity. The best results were achieved with samples containing 5% and 7.5% carbon-fiber content, where strength increased by 22.5% and the modulus of elasticity by 30.5%. Longer fibers (150 μm) showed higher strength than shorter ones (100 μm) but lower toughness and ductility. However, porosity increased with higher fiber concentrations, negatively affecting mechanical properties [15,19]. The choice of carbon-fiber type significantly influences the mechanical properties of composites. T300 fibers, as shown by the research of Ramírez-Herrera et al., with their rough surface texture, ensure stronger bonding with the matrix, reducing the risk of delamination. On the other hand, TZ300 fibers, despite their high graphitization, exhibit lower stability after high-temperature processing [26].

The integration of short-fiber and carbon composites in modern manufacturing technologies allows materials to be effectively tailored to specific requirements. The combination of numerical simulations, experimental data, and advanced technological processes leads to the development of components capable of meeting the challenges of modern industries, such as brakes, clutches, and aerospace structures, where high durability, strength, and reliability are essential [15,17,18,19,20,21,22,25,26].

### 2.2. Hybrid Graphene and Carbon Composites

Hybrid graphene and carbon composites represent a significant category of modern materials with high potential for industrial applications. These composites offer a combination of high strength, low weight, and excellent mechanical properties, which are fundamentally influenced by the quality of the interface between the fibers and the matrix. Various innovative technologies are employed to enhance these properties, including the application of graphene nanoplatelets, fluorinated sizing agents, and other structural modifications [27,28].

**Figure 4:**
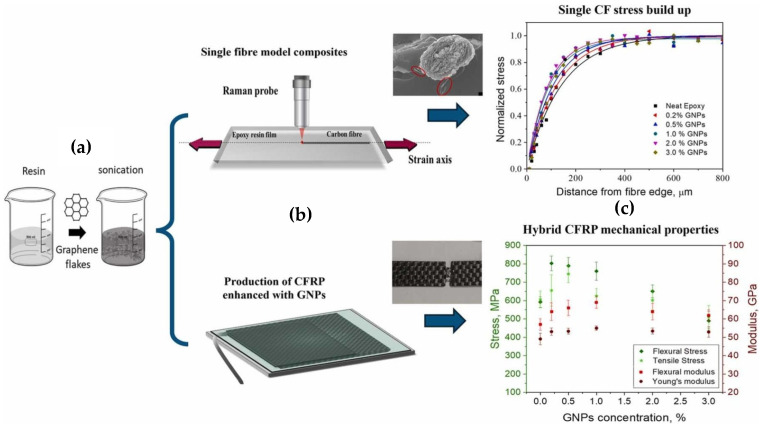
The reinforcement mechanism of hybrid CFRP composites: (**a**) Raman analysis of stress in single-fiber composites; (**b**) production of CFRP with graphene nanoparticles (GNPs); (**c**) mechanical properties of hybrid composites with GNPs [29].

One of the primary methods for improving interfacial properties, as demonstrated by the research of Österle et al., is the integration of graphene nanoplatelets (GNPs), which offer exceptional mechanical and electrical properties. Figure 4 provides an overview of the reinforcement mechanism of these hybrid CFRP composites. It illustrates Raman analysis of stress in single-fiber composites, the production process of CFRP enhanced with graphene nanoparticles (GNPs), and the subsequent improvement in the mechanical properties of the composites. Research has shown that an optimal concentration of GNPs in the epoxy matrix (e.g., 0.5–1.0 wt%) leads to an increase in flexural strength by up to 35% and interlaminar shear strength (ILSS) by 45%. The uniform distribution of GNPs within the matrix enables better stress transfer between the carbon fibers and the matrix, thereby minimizing the risk of delamination [27].

**Figure 5:**
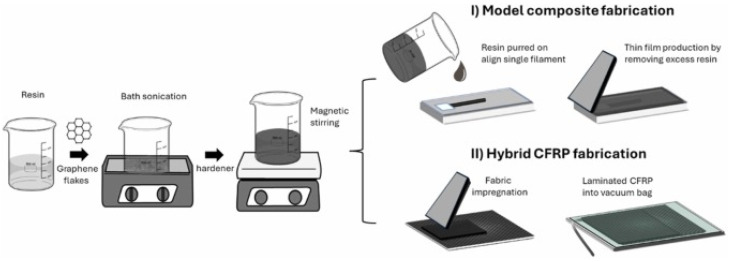
Schematic representation of the production process of (**I**) model composite; (**II**) a hybrid CFRP [29].

The production process of the model composite and hybrid CFRP is detailed in Figure 5, which illustrates the individual steps, from the sonication of graphene flakes in the resin and magnetic stirring with the hardener to the impregnation of fabrics and lamination of CFRP in a vacuum bag. These steps are essential to achieving a homogeneous composite structure. Fluorine-enriched sizing agents, which minimize surface defects on carbon fibers and improve their wettability with resin, play a crucial role in this process. Experimental results have shown that the use of fluorinated agents increases interlaminar shear strength (ILSS) by 15.79% and reduces moisture absorption, which is critical for the long-term durability of the material under demanding conditions [28].

**Figure 6:**
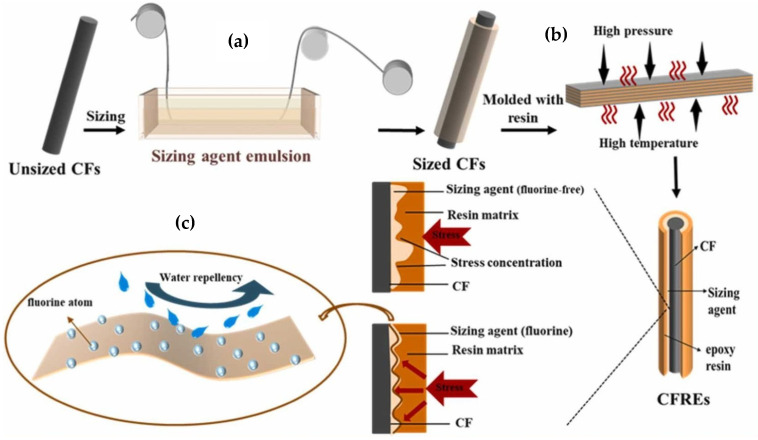
The process of carbon-fiber (CF) treatment and CF composite production: (**a**) application of sizing agent; (**b**) molding with epoxy resin; (**c**) mechanism of moisture protection and stress concentration reduction [30].

The process of carbon-fiber (CF) treatment and CF composite production, as illustrated in Figure 6, includes the application of a sizing agent to the fiber surface, subsequent molding with epoxy resin, and mechanisms for moisture protection. The schematic details how the sizing agent provides hydrophobicity and reduces stress concentration, leading to improved mechanical properties of the composites.

The combination of nano- and microparticles, such as carbon nanotubes (CNTs) or graphene layers, offers an additional approach to enhancing toughness and interlaminar properties. For instance, the application of polydopamine (PDA) as a coupling medium between carbon fibers and nanoparticles improves adhesion between the matrix and the fibers. Mechanisms such as nanotube pull-out or their sliding friction significantly enhance the material’s ability to resist cracking [31].

**Figure 7:**
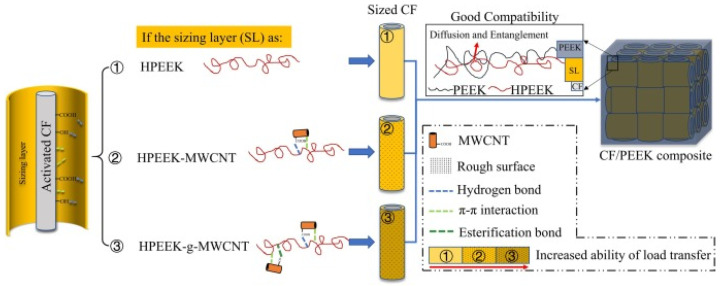
Schematic of the proposed interaction mechanism for modified CF/PEEK composites [32].

As illustrated in Figure 7, the proposed interaction mechanism between carbon fibers (CF) and a polyetheretherketone (PEEK) matrix involves the use of various types of sizing layers, including modifications with multi-walled carbon nanotubes (MWCNTs) and low-melting polyaryletherketones (LMPAEKs). The figure depicts three different configurations of sizing layers: pure HPEEK, HPEEK modified with MWCNTs, and HPEEK grafted with MWCNTs. Each configuration demonstrates different mechanisms contributing to improved compatibility between the fibers and the matrix. These mechanisms include hydrogen bonding, π–π interactions, esterification bonds, and enhanced diffusion capacity.

LMPAEK not only provides better adhesion but also increases resistance to crack propagation, significantly improving the toughness of the composites. The figure also highlights the importance of the rough fiber surface, which enables stronger mechanical interlocking with the matrix, resulting in higher mechanical stability, delamination resistance, and improved load transfer. The outcome is a material with greater strength and thermal stability, suitable for automotive and aerospace applications where mechanical and thermal properties are critical [29,30,31,32].

**Figure 8:**
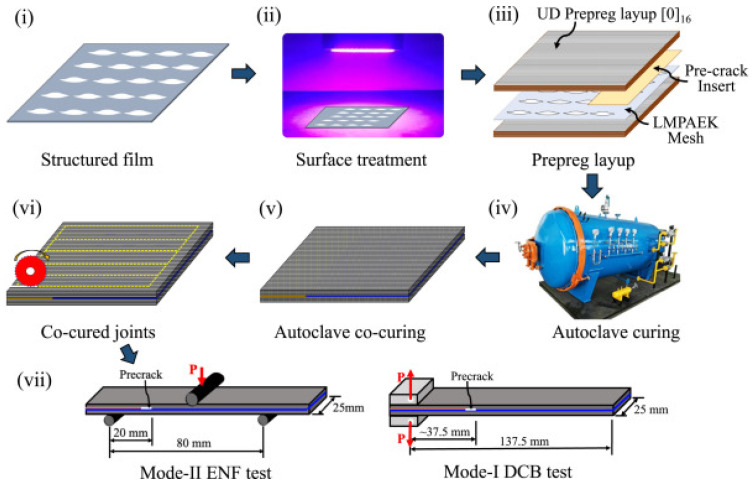
The manufacturing process and testing of CFRP composites: (**i**) structured film; (**ii**) surface treatment; (**iii**) prepreg preparation; (**iv**) autoclave curing; (**v**) co-curing of joints; (**vi**) final lamination; (**vii**) mechanical tests (Mode-II ENF and Mode-I) [33].

The manufacturing process and testing of CFRP composites are detailed in Figure 8, which outlines the steps from the preparation of the structured film and surface treatment, through prepreg preparation and autoclave curing, to final lamination and mechanical tests (Mode-II ENF and Mode-I). The process begins with the structured film and surface treatment, optimizing surface properties for subsequent lamination. During autoclave curing and co-curing of joints, perfect bonding between layers is achieved, ensuring a homogeneous laminate structure.

Mechanical tests, such as Mode-II ENF and Mode-I DCB, validate the properties of the final material, particularly its resistance to cracking and its ability to withstand dynamic loads. This scheme highlights the importance of each phase in ensuring the desired properties of the composites, including strength, moisture resistance, and thermal stability. The synergistic effect of these innovative approaches results in the production of materials with enhanced strength, extended lifespan, and improved properties for industrial applications under extreme conditions [27,28,29,31,33].

### 2.3. Hybrid Reinforcement and Interlayers

Reinforcement of composites is a critical aspect in enhancing their mechanical properties, toughness, and resistance to crack propagation. Hybrid reinforcement combines multiple technologies and materials that synergistically improve composite performance while minimizing adverse effects on their structure. This approach is particularly valuable in applications where toughness and mechanical resilience are paramount, such as in the aerospace and automotive industries [29,34].

One method of hybrid reinforcement involves the addition of thermoplastic interlayers, which serve as barriers between composite layers. Low-melting polyaryletherketones (LMPAEKs) are examples of thermoplastics used to enhance interlaminar adhesion and crack resistance. When processed into specific hollow structures, they optimize epoxy resin flow during manufacturing and increase the contact area between the matrix and fibers. These structures often undergo UV activation to enhance interaction between the thermoplastic and the epoxy matrix. The result is a significant increase in interlaminar fracture energy and toughness, even at elevated temperatures [34].

Hybrid reinforcement also frequently includes nanomaterials, such as carbon nanotubes (CNTs) or multi-layer graphene particles (mG). These fillers are effective in improving stress transfer and fracture resistance. Mechanisms such as nanotube pull-out or sliding contribute to a notable increase in interlaminar toughness. Mode-I tests revealed a 101% increase in fracture energy with graphene, while Mode-II tests showed a 154% increase with CNTs. These improvements result from enhanced interaction between fibers and the matrix [29,35].

Another approach to enhancing interlaminar toughness is the use of carbon-fiber (CF) layers in nature-reinforced composites (FFRCs). Research by Zhang et al. in the study Experimental and numerical investigation into interlaminar toughening effect of chopped fiber-interleaved flax fiber reinforced composites experimentally and numerically analyzed factors such as carbon-fiber type, length, and areal density of the reinforcement to optimize mechanical properties. Double cantilever beam (DCB) tests showed that the best results were achieved with carbon-fiber reinforcement of 5 mm in length and 25 g/m^2^ areal density. The critical value of specific fracture energy GIC reached 2.322 kJ/m^2^, an 80.7% improvement. Toughening mechanisms included fiber bridging, fiber fibrillation, and multi-layered failure. Numerical simulations using the cohesive zone model (CZM) demonstrated good agreement with experimental data, providing guidance for designing eco-friendly composites [36].

An innovative approach to hybrid reinforcement involves the use of amine-functionalized graphene (ADG-NH_2_) in combination with an epoxy matrix, leading to significant improvements in interlaminar and mechanical properties. Research by Xu et al. demonstrated that ADG-NH_2_ acts as an effective coupling agent, enhancing the interaction between carbon fibers and the epoxy matrix. This functionalization ensures better stress transfer and significantly reduces the likelihood of delamination. Tests showed that adding 0.5 wt% ADG-NH_2_ to the epoxy matrix increased flexural strength by 33%, interlaminar shear strength (ILSS) by 27%, and toughness by 28% [35].

**Figure 9:**
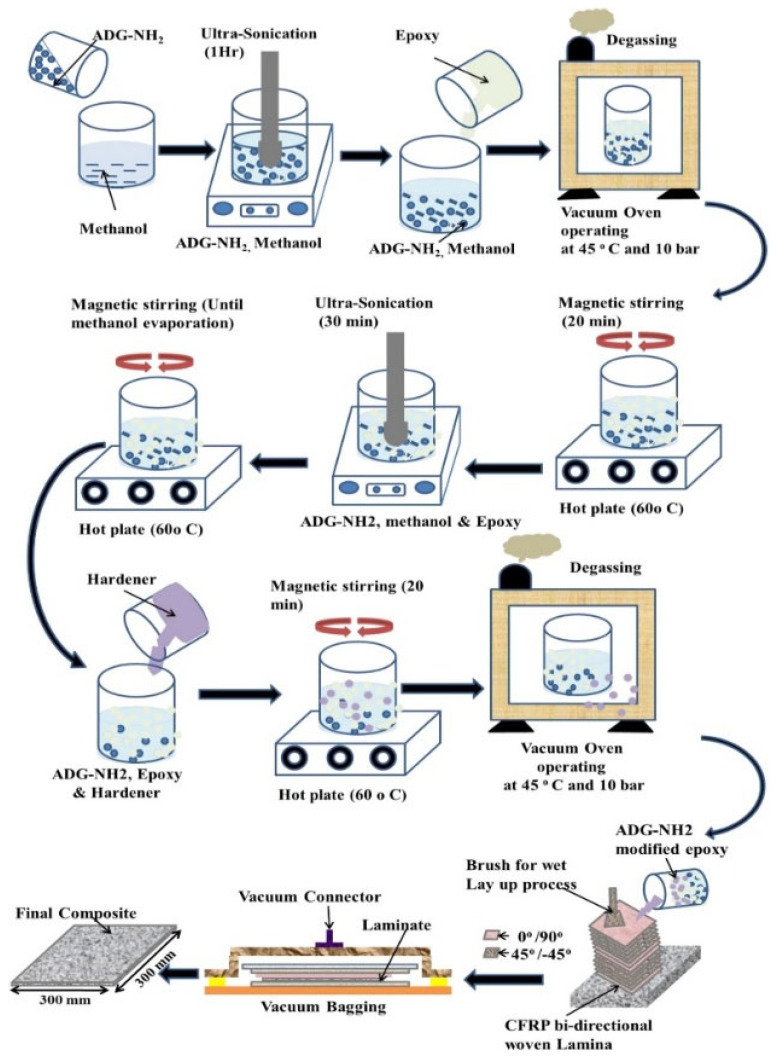
The preparation process of CFRP composites with ADG-NH_2_-modified epoxy resin: ultrasonic dispersion, mixing, degassing, lamination, and vacuum pressing [29].

As illustrated in Figure 9, the preparation process of CFRP composites with ADG-NH_2_-modified epoxy resin involves several steps. Initially, ADG-NH_2_ is dispersed in methanol using ultrasonic sonication, followed by mixing with epoxy resin and the evaporation of methanol on a hot plate. Subsequently, a hardener is added to the mixture, which undergoes magnetic stirring and degassing in a vacuum oven. After final adjustments, the modified epoxy resin is applied to biaxial CFRP fabric, which is then laminated and vacuum-pressed. This intricate process ensures the homogeneous distribution of nanomaterials within the matrix, leading to enhanced mechanical properties [29,35].

Hybrid reinforcement also addresses challenges such as unstable crack propagation behavior and limited compatibility with certain manufacturing processes. The introduction of precisely engineered interlayers and nanomaterials optimizes mechanical properties without increasing the thickness or weight of the composites. These technologies represent a step forward in designing advanced materials that meet the stringent requirements of the aerospace and automotive industries [29,35]. Hybrid reinforcement and interlayers thus provide an effective approach to improving toughness, crack propagation resistance, and overall mechanical performance of composites. These advanced techniques enable the creation of materials capable of withstanding demanding conditions without compromising their integrity and performance [29,34,35,36].

## 3. Surface Treatments and Fiber Reinforcement Interfaces: Optimization of Carbon-Fiber Interfaces Using Polydopamine

Surface treatments of carbon fibers are essential for improving adhesion between the fibers and the epoxy matrix. The use of polydopamine (PDA) enhances the surface roughness and polarity of the fibers, resulting in better wettability and interfacial strength. Coatings with ceramic materials, such as silicon dioxide or silicon carbide, further improve the oxidation resistance of the fibers, extending their lifespan at elevated temperatures. These treatments also optimize stress transfer within the material, thereby increasing the overall mechanical stability of the composites [1,3,5,6].

The quality of the interface between carbon fibers and the epoxy resin plays a pivotal role in determining the mechanical properties and durability of composite materials. Improving adhesion between fibers and the matrix can lead to significant enhancements in strength, shear load resistance, and composite lifespan. One innovative solution is the application of a polydopamine (PDA) coating, which creates effective interfacial layers and optimizes compatibility between the individual components [1,2].

**Figure 10:**
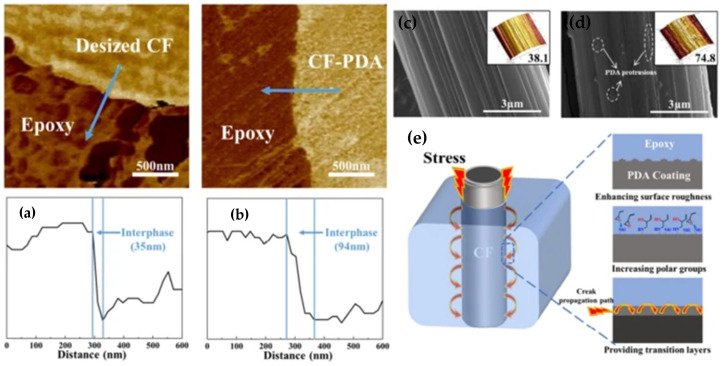
Mapping of force modulation via AFM and hardness distribution at the composite interface: (**a**,**c**) desized CF; (**b**,**d**) CF-PDA9; (**e**) Mechanism of interfacial reinforcement with PDA coating [6].

As shown in Figure 10, the PDA coating significantly alters the interface between carbon fibers (CFs) and the epoxy matrix. Compared to desized fibers (CFs without coating), CFs with PDA coating exhibit a substantially wider interphase (94 nm compared to 35 nm), as confirmed by AFM force modulation mapping and hardness distribution at the composite interface. The increased thickness and surface roughness of the fibers improve interaction with the matrix, leading to better stress transfer and enhanced delamination resistance. The PDA coating also increases the polarity of the fibers, resulting in improved wettability and higher interfacial strength [1,3].

The polydopamine coating enhances the surface roughness of carbon fibers, improving adhesion with the epoxy resin. Additionally, this coating increases fiber polarity, leading to better wettability and, consequently, higher interfacial strength. Experimental studies have shown that the PDA coating reduces surface defects on the fibers and absorbs additional loads, significantly reducing the risk of delamination and cracking under dynamic loads. These properties are particularly beneficial in applications where composites are subjected to cyclic loading, such as in aerospace [1,3].

Controlled PDA coating thickness has been identified as a key factor influencing mechanical properties. During a nine-hour polymerization process of PDA on the fibers, tensile strength increased by 18.64%, and interlaminar shear strength improved by 35.06%. These results demonstrate that proper tuning of the thickness and composition of the PDA layer is crucial for optimizing compatibility between fibers and the epoxy matrix [6]. Furthermore, the PDA coating allows for uniform stress transfer between the fibers and the matrix, eliminating stress concentration at specific interfacial points. This transition mechanism significantly enhances the overall strength and resistance of the composite to dynamic loading.

The combination of PDA coating with sophisticated surface treatments represents a significant step forward in improving the performance of composite materials [4,5]. Research findings indicate that optimizing the interface between carbon fibers and the epoxy matrix with PDA coating not only enhances mechanical properties but also extends the lifespan of composites, making these materials suitable for a wide range of demanding applications [5,6].

### Coating Carbon Fibers for Oxidation Resistance

Carbon fibers are a vital component of advanced composites, known for their exceptional properties such as high strength, low weight, corrosion resistance, and high modulus of elasticity. However, research by Fu et al. highlights a significant limitation of carbon fibers—their low oxidation resistance at high temperatures. Oxidation begins at around 750 K in air, significantly restricting their application in high-temperature environments. To enhance their oxidation resistance, various surface treatments and coating techniques are employed to protect the fibers while maintaining their mechanical properties [37].

One of the most effective methods for improving oxidation resistance is the application of ceramic coatings, such as silicon carbide (SiC), silicon dioxide (SiO_2_), or aluminum oxide (Al_2_O_3_). These coatings form a barrier between the carbon fibers and the surrounding environment, preventing oxygen infiltration and minimizing the oxidation process. For example, SiC coatings applied via chemical vapor deposition (CVD) have demonstrated significant improvements in the oxidation resistance of carbon fibers, although with a slight reduction in fiber strength. SiC coatings are particularly effective at temperatures above 1370 K, providing long-lasting protection against degradation [37,38].

**Figure 11:**
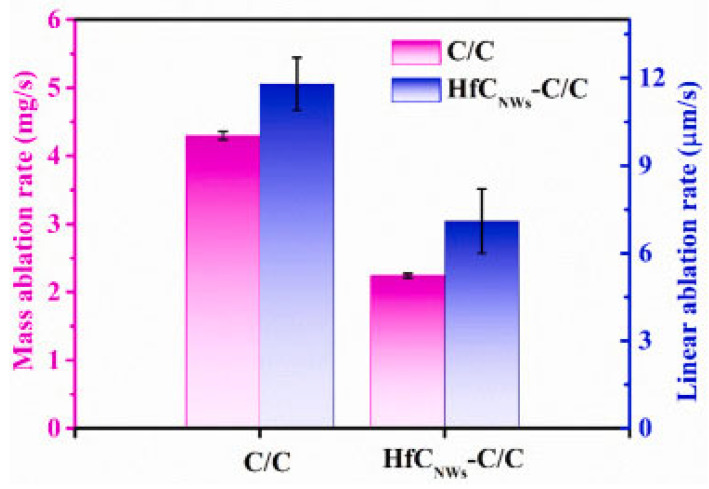
Comparison of ablation rates: C/C composites vs. HfC_NWs_-C/C composites (mass ablation and linear ablation) [36].

As shown in Figure 11, HfC_NWs_C/C composites demonstrate significantly lower rates of mass and linear ablation compared to traditional C/C composites. This characteristic is critical for applications in high-temperature environments, where materials are exposed to intense thermal loads and oxidation. The effectiveness of ceramic coatings like HfC lies in their ability to provide superior protection against thermal degradation while maintaining the structural integrity of the material under extreme conditions [37,38].

In addition to ceramic coatings, multi-layer gradient coatings, such as PC//SiC//Si combinations, have proven effective in addressing oxygen penetration through microdefects in the coating. The gradual change in coating composition reduces interlayer stress and enhances overall oxidation resistance. These gradient coatings also minimize crack formation and defects, ensuring longer service life in extreme conditions [24,37].

The sol–gel method offers a simpler and more cost-effective alternative to CVD techniques for applying oxide coatings. Silica (SiO_2_) and alumina (Al_2_O_3_) coatings prepared using this method have improved the oxidation resistance of carbon fibers, albeit not to the extent of SiC coatings. However, sol–gel coatings are prone to microdefects and pores, which allow partial oxygen penetration, reducing their protective effect over time. To enhance the efficacy of sol–gel coatings, it is recommended to control the atmosphere during pyrolysis or incorporate silica nanoparticles to minimize defect formation [38].

C/SiC ceramic composites are promising materials for turbine engines due to their low weight and high-temperature resistance, but their primary challenge is protecting carbon fibers from oxidation at temperatures above 400 °C. Studies have shown that Si layers applied by plasma spraying (PS) can significantly reduce carbon fiber oxidation by forming a protective SiO_2_ layer with a low oxygen diffusion coefficient. Although the Si layer enhances oxidation resistance, prolonged oxidation leads to an increase in cracks and pores, potentially causing delamination. Research also indicates that a 150 μm Si layer provides better oxidation resistance than a 200 μm layer. These findings are crucial for developing more durable materials for long-term use in high-temperature turbine applications [39].

Beyond oxidation protection, improving adhesion between carbon fibers and the matrix in polymer composites is another key aspect of enhancing material properties. Research by Liu et al. demonstrates that low interfacial adhesion strength limits the effective load transfer from the matrix to the fibers, reducing the overall mechanical properties of composites. Methods such as anodization, plasma treatments, and oxidations increase the surface energy of fibers and improve their interaction with the matrix. Recent studies have also explored the use of rare earth elements, such as praseodymium (Pr), for carbon fiber surface treatments. Rare earth surface treatments increase fiber roughness and create oxygen-containing functional groups, enhancing adhesion and improving composite mechanical properties. After treatment with rare earth elements, the interlaminar shear strength (ILSS) of composites increased by 8.5%, with gamma irradiation further improving it by 13.1% [40].

Similarly, other surface treatments like grafting acrylic acid onto carbon fibers have been used to improve adhesion with epoxy matrices. Research by Wang et al. shows that this technique, especially when combined with γ-ray irradiation and oxidation–reduction (KMnO_4_/H_2_SO_4_), effectively increases surface polarization, wettability, and matrix interaction. This method enhances oxygen content and functional groups on the fiber surface, resulting in better adhesion and a significant increase in ILSS [41].

Another effective method for improving adhesion between carbon fibers and epoxy matrices is hydrogen peroxide treatment in supercritical water. Research by Xiao et al. reveals that this method increases the number of oxygen-containing functional groups on the fiber surface, significantly improving fiber–matrix interaction. AFM and XPS analyses confirm increased surface roughness and oxygen functional group content. These modifications promote chemical bonding between the fibers and the epoxy matrix, leading to improved ILSS in composites. Hydrogen peroxide treatment in supercritical water thus proves to be an efficient approach to enhancing interfacial properties in carbon–polymer composites [34].

The practical application of coated carbon fibers requires an optimal balance between their mechanical properties and oxidation resistance. While CVD methods provide the best protection, they come at the cost of reduced fiber strength and higher production costs. On the other hand, sol–gel methods offer simpler application and lower costs, making them suitable for broader industrial use. Combining these techniques, such as applying gradient multi-layer coatings with sol–gel layers, can produce a synergistic effect, ensuring high oxidation resistance and preserving the mechanical integrity of fibers [37,38].

Research also highlights that carbon-fiber surface treatments, such as sandblasting before electroplating, can significantly improve adhesion between composite surfaces and metallic layers like copper. This process, which increases surface roughness and exposes carbon fibers, has demonstrated up to a tenfold increase in peel strength, suggesting a novel approach to developing composites with mechanically and thermally resistant metallic layers, particularly for applications such as cryogenic liquid hydrogen storage tanks [35]. These findings demonstrate that modern coating technologies expand the applicability of carbon fibers in extreme environments, such as high-temperature aerospace and energy industry applications. Oxidation protection and improved fiber–matrix adhesion are key factors that contribute significantly to the long-term performance and reliability of composite materials [16,24,37,38,42,43,44,45].

## 4. Tribological and Fatigue Properties of Composites: Tribological Properties of Epoxy Composites

Epoxy composites reinforced with carbon fibers and nanoparticles exhibit significantly improved tribological properties, such as low friction and high wear resistance. Self-lubricating systems with microcapsules gradually release lubricant, ensuring uniform surface protection and reducing wear rates. Under cyclic loading, hybrid composites made from carbon and glass fibers demonstrate higher delamination resistance, while nondestructive methods, such as infrared thermography, help monitor the development of microcracks and extend the material’s lifespan [32,37,46,47].

Tribological properties, such as friction and wear, play a crucial role in the performance of epoxy composites in applications involving repeated mechanical contact and dynamic loading. The addition of specialized reinforcements, such as carbon fibers and silicon dioxide nanoparticles, significantly enhances the wear resistance of composites and reduces friction, thereby extending their service life [32,46,48].

**Figure 12:**
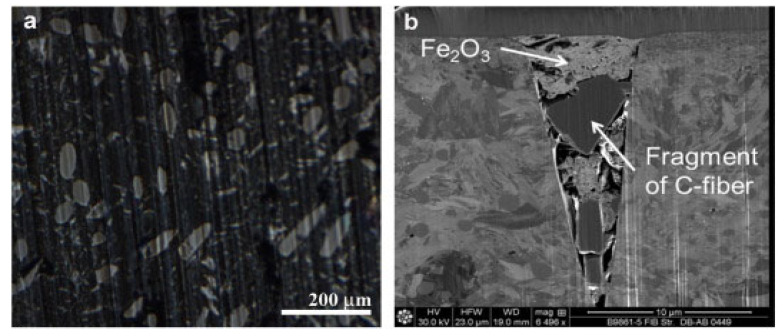
Microstructural analysis of composites: (**a**) distribution of carbon fibers within the matrix; (**b**) carbon fiber fragmentation and Fe_2_O_3_ deposits [32].

As illustrated in Figure 12, microstructural analysis confirms the uniform distribution of carbon fibers within the matrix (part a) and shows fiber fragmentation alongside Fe_2_O_3_ deposits (part b). This combination of reinforcements enhances the tribological properties of the composites, making them suitable for dynamically loaded applications [32,46,48].

Carbon fibers serve as primary contact points that absorb mechanical loads and reduce friction. Simultaneously, silicon dioxide nanoparticles form thin protective films on the surface of composites, minimizing abrasive wear. These films create a barrier that prevents heat penetration into the matrix, protecting the material from thermal degradation. Experiments have shown that under moderate loads, the protective films remain stable; however, at high load values (pv—the product of pressure and speed), the films deteriorate, leading to increased wear [32,48].

The combination of carbon fibers and silicon dioxide nanoparticles exhibits a synergistic effect. Carbon fibers enhance the strength and stiffness of the composites, while the nanoparticles reduce friction and wear under low and medium loads. This synergistic effect makes these composites ideal for mechanisms such as bearings, gears, and drive systems, where long-term reliability and durability are critical [32,46].

Structural observations reveal that local heating of carbon fibers during friction can reach high temperatures, causing tribofilm degradation and oxidation of the contact surfaces. Despite these challenges, epoxy composites reinforced with carbon fibers and silicon dioxide nanoparticles demonstrate significantly higher resistance to abrasive wear compared to conventional polymer materials [46,48].

Findings suggest that optimizing the content of carbon fibers and silicon dioxide nanoparticles enables the development of epoxy composites that offer a balanced performance in terms of strength, low friction, and wear resistance. These properties make them well-suited for a wide range of industrial applications where reliability under mechanical stress is essential [32,46].

### 4.1. Self-Lubricating Systems with Microcapsules

Self-lubricating systems based on polytetrafluoroethylene (PTFE) and aramid-fiber textile composites are widely used in industry due to their low maintenance requirements, excellent tribological properties, and high mechanical durability. These systems are particularly applied in aerospace and automotive industries, where minimizing friction and wear is critical for ensuring long-term reliability. However, the performance of these materials under high temperatures and loads is often limited, leading to increased wear and reduced service life [47].

An innovative solution to improve the tribological properties of these composites is the incorporation of microcapsules filled with liquid lubricants. Research by Xu et al. demonstrated that microcapsules such as polyethersulfone/polymethylphenylsiloxane (PES/PMPS) provide lubrication during the initial stages of friction and gradually release lubricant under increased load or temperature. This mechanism creates a stable and uniform transfer film on the material’s surface, reducing wear and enhancing resistance to damage.

**Figure 13:**
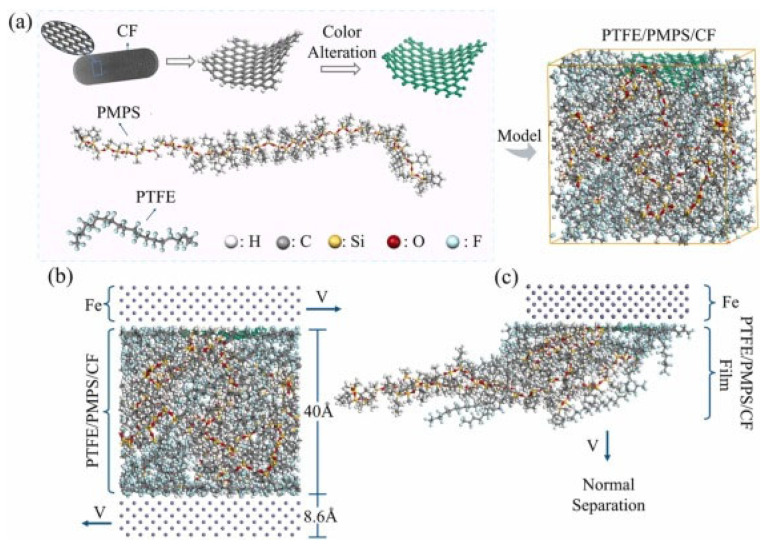
Modeling the interaction of the PTFE/PMPS/CF system: (**a**) chemical structure and coloring of CF; (**b**) simulation of molecular arrangement; (**c**) normal layer separation [47].

As shown in Figure 13, the chemical structure of the PTFE/PMPS/CF system was analyzed through modeling the interactions of individual components. Part (a) illustrates the chemical structure of the system, including the modified carbon fibers. Parts (b) and (c) depict molecular arrangements and simulations of layer separation, demonstrating the mechanism of transfer film formation. This system offers significantly improved wear protection while reducing friction under high-load conditions.

Due to these properties, the PTFE/PMPS/CF system appears to be a promising solution for applications requiring advanced tribological characteristics and high resistance to mechanical damage [47].

Research by Xu et al. and Fu et al. highlights that, in addition to lubricating microcapsules, carbon fibers are also incorporated into composites to enhance mechanical stability and wear resistance. Carbon fibers act as a structural framework that transfers load while improving the efficiency of the transfer film. Tribological tests revealed that the combination of PES/PMPS microcapsules and carbon fibers creates a synergistic effect, significantly reducing the coefficient of friction and wear rate. These composites achieved a coefficient of friction of 0.076 and a wear rate of 0.48 × 10^−8^ mm^3^/(N·m), representing a 17% reduction in friction and a 24% reduction in wear compared to standard liners [37,47].

**Figure 14:**
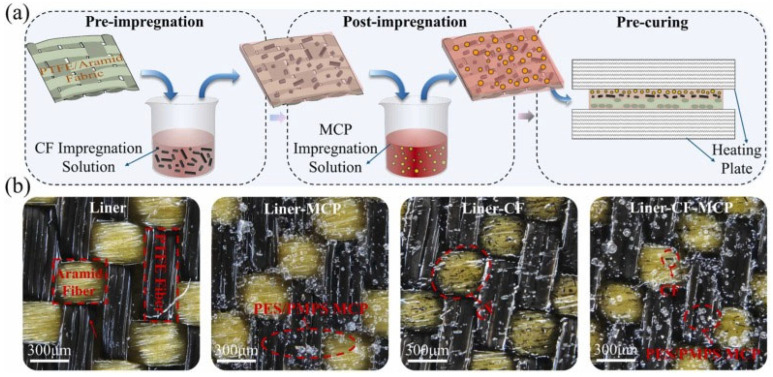
The impregnation process and composite structure: (**a**) preimpregnation, postimpregnation, and precuring of CF/aramid fibers; (**b**) microstructural layer analysis (liner, MCP, CF, CF-MCP) [36].

As illustrated in Figure 14, the impregnation process and microstructural layer analysis demonstrate the effective distribution of carbon fibers and microcapsules, contributing to the formation of a mixed transfer film and increasing the system’s durability.

The improvement mechanism lies in the formation of a mixed transfer film composed of PTFE, PMPS, and carbon fibers. This film not only reduces adhesive friction but also prevents localized overheating of the surface, thereby enhancing the overall durability of the system. Scanning electron microscopy (SEM) analysis revealed a homogeneous distribution of the transfer film, contributing to its stability under high temperatures and loads [36,47].

The combination of lubricating microcapsules and carbon fibers is thus an effective solution for enhancing the tribological properties of PTFE/aramid textile composites. This technology offers long-term stability and high performance in demanding conditions, making it attractive for a wide range of industrial applications, such as bearings, tribosystems, and components operating in high-temperature and high-load environments. The improved properties of these composites also reduce maintenance requirements and extend the lifespan of systems, leading to increased efficiency and reliability [37,47].

### 4.2. Fatigue Properties and Cyclic Loading of Composites

Fatigue properties of composites play a critical role in their application in structures subjected to cyclic loading, such as aerospace components, wind turbines, or the automotive industry. Materials like carbon-fiber-reinforced polymers (CFRPs) and hybrid composites made from carbon and glass fibers exhibit various damage mechanisms that require detailed analysis and optimization for long-term use [25,49].

**Figure 15:**
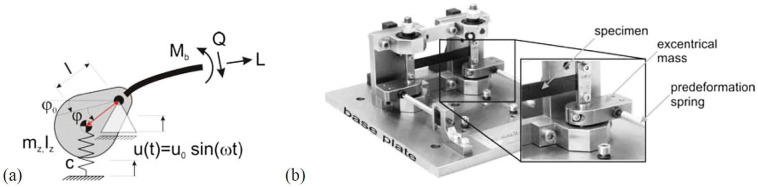
Experimental setup for dynamic testing: (**a**) schematic of harmonic motion with predeformation; (**b**) mechanical system with an eccentric mass and predeformation spring [25].

As illustrated in Figure 15, the experimental setup includes a system with an eccentric mass and a predeformation spring, enabling the simulation of cyclic loading and evaluation of material fatigue resistance.

CFRP composites are renowned for their high strength and resistance to cyclic loading. For instance, the load-bearing structures of wind turbine blades must endure over a billion cycles without significant damage. Traditional fatigue tests at low frequencies require extended durations to obtain results, which is why high-frequency methods (>150 Hz) are used for studying very high cycle fatigue (VHCF). Specialized testing systems minimize sample heating and provide precise data on the degradation of fibers and interfiber bonds. The surface treatment of carbon fibers significantly impacts the delamination resistance and interlaminar damage tolerance of CFRP [25].

Hybrid composites, such as laminates made of carbon and glass fibers, face distinct challenges under cyclic loading. Fiber wrinkles formed during manufacturing weaken the structure and lead to premature material failure. Studies show that these wrinkles can reduce compressive cyclic strength by up to 50%, with delamination and microcracking appearing in the early stages of cycles. Nondestructive methods like acoustic emission (AE) and infrared (IR) thermography allow for monitoring these defects. IR thermography identifies microcracks based on thermal anomalies, while AE detects early delamination activities, enhancing the accuracy of damage diagnostics [49].

These findings, supported by research from Li et al. and Anvari et al., highlight the importance of developing new technologies to optimize the fatigue properties of composites. Proper surface treatments, minimizing defects during manufacturing, and deploying advanced diagnostic tools ensure the long-term reliability of these materials under demanding industrial conditions [25,49].

## 5. Thermal and High-Temperature Resistance of Composites: Fire-Resistant Coatings with Hexaphenoxycyclotriphosphazene and Expanded Graphite

Epoxy composites with fire-resistant coatings, such as hexaphenoxycyclotriphosphazene (HPCTP) and expanded graphite (EG), demonstrate enhanced thermal stability and flame resistance. The addition of high-temperature reinforcements, such as carbon fibers and ceramic fillers, further improves their strength and stability at temperatures above 300 °C. Under dynamic loading combined with high temperatures, properly designed composites resist delamination and deformation, making them suitable for extreme environments [10,11,12].

Fire-resistant properties of composite materials are crucial in applications prioritizing safety, such as construction, transportation, or industrial environments. An effective solution involves using specialized coatings that combine HPCTP and EG. These coatings offer a synergistic effect, reducing material flammability and increasing thermal stability [10,11,12].

**Figure 16:**
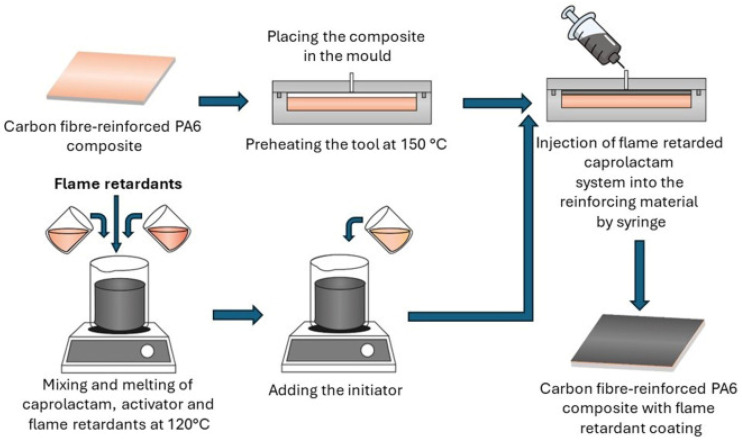
The composite manufacturing process [10].

As shown in Figure 16, the composite manufacturing process involves mixing caprolactam, activators, and fire-resistant additives at 120 °C, followed by injection into the composite material. This method ensures uniform application of fire-resistant layers, which act as a thermal shield and reduce flame spread. Experimental results indicate a 68% reduction in peak heat release rate and a 29% decrease in total heat release compared to materials without coatings. This technology is particularly effective for thermoplastic composites, where the combination of safety and recyclability is critical [10,12].

Hexaphenoxycyclotriphosphazene contributes to the formation of a protective film in the gas phase, while expanded graphite acts as a barrier in the condensed phase. This synergistic effect enhances the fire-resistant properties of the composite while maintaining its mechanical performance. A coating only 0.5 mm thick applied to the surface of carbon-fiber-reinforced polyamide composites reduces the peak heat release rate by 33% and the total heat release by 37%, demonstrating the effectiveness of this technology [10,11].

**Figure 17:**
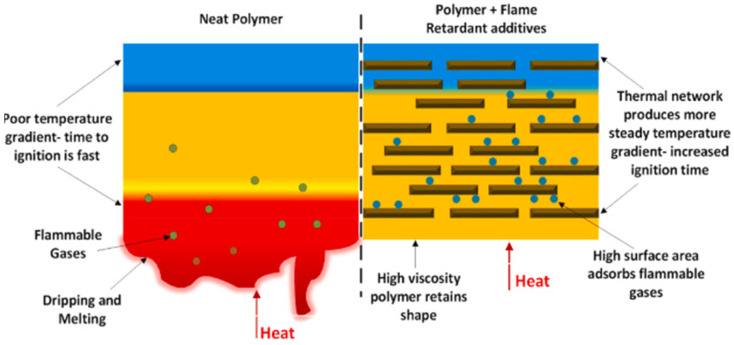
The impact of the extensive surface area of flame-retardant additives on the combustion properties of polymers [46].

As shown in Figure 17, the addition of flame-retardant additives creates a thermal network that stabilizes the temperature gradient and delays ignition. While pure polymer burns quickly and releases flammable gases, the modified polymer with HPCTP and EG retains its shape and significantly limits flame spread. This property minimizes the risk of deformation or degradation during a fire [46].

In addition to flame protection, these coatings enhance the high-temperature resistance of composites, reducing the risk of deformation or degradation during fires. Their application is also cost-effective, as the low material requirements enable straightforward implementation across a wide range of industrial products. Due to these properties, HPCTP and EG are ideal additives for producing safe and durable materials [10,12].

Research results confirm that the combination of HPCTP and EG in fire-resistant coatings represents a significant advancement in improving the safety of composite materials. This technology provides reliable fire protection without compromising the performance or mechanical properties of the composite, making it suitable for the most demanding industrial applications [10,37,46].

### 5.1. High-Temperature Resistance and Strength of Epoxy Composites

Composites reinforced with carbon fibers and nanomaterials represent advanced materials with exceptional mechanical and thermal properties, making them ideal for applications in extreme conditions. These materials are designed to withstand high temperatures and intense mechanical loads, which is particularly critical in the aerospace and space industries. The development of these composites involves the integration of high-performance nanostructures and specialized technological modifications to ensure their durability and stability [30,38,50].

**Figure 18:**
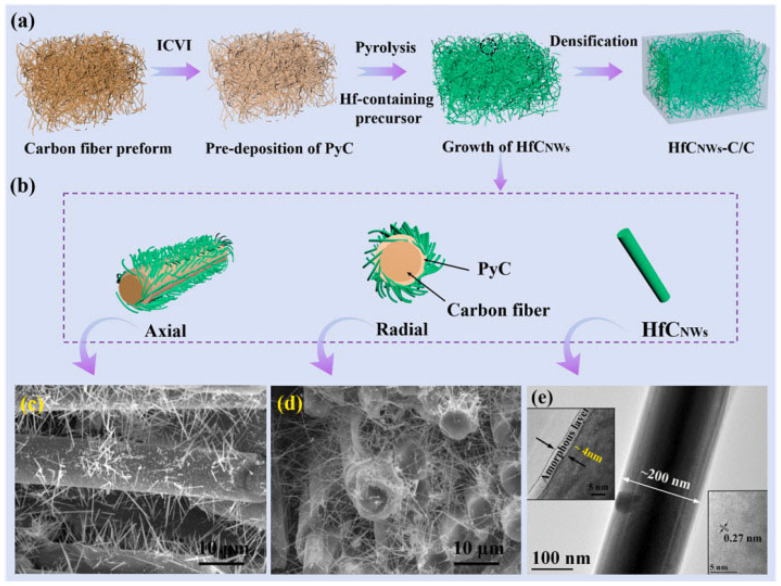
(**a**,**b**) Schematic diagram of the process of HfC_NWs_-C/C; Microstructure of HfC_NWs_ (**c**,**d**) SEM images of the as-prepared HfC_NWs_ in carbon fiber preforms; (**e**) TEM image, inset on the left shows the edge area of the HfC_NWs_, while inset on the right exhibit the HRTEM image [51].

As shown in Figure 18, the preparation process for HfC nanowires (HfCNWs) involves multiple steps, including pyrolysis, growth of HfCNWs, and strengthening of the carbon matrix. This process results in structurally efficient nanowires that act as reinforcements within the carbon matrix, promote graphitization, and enhance interlaminar shear strength (ILSS). SEM and TEM analyses (parts c, d, and e) reveal the homogeneous distribution of HfCNWs in carbon fiber preforms and their structural integrity at the nanometric level [36].

The addition of one-dimensional nanostructures, such as HfC nanowires, significantly improves the mechanical properties of carbon composites under extreme temperatures exceeding 2000 °C. HfC nanowires enhance out-of-plane compressive strength (OCS) and flexural strength (FS) while reducing ablation rates, which is critical for applications such as rocket nozzles and hypersonic aircraft. Their combination of properties, including high resistance to thermal shocks, reduced wear rates, and exceptional strength, makes these composites invaluable for highly demanding environments [30,38].

The manufacturing process for nanowire-reinforced composites involves the application of chemical vapor infiltration (ICVI), which enables the formation of a three-dimensional nanowire structure within carbon preforms. These structures improve the mechanical bonding between carbon fibers and the matrix, thereby enhancing the material’s overall mechanical stability. Testing has demonstrated that these composites exhibit significantly higher resistance to high temperatures and a reduction in linear ablation by more than 20% compared to traditional carbon composites [36,38].

**Figure 19:**
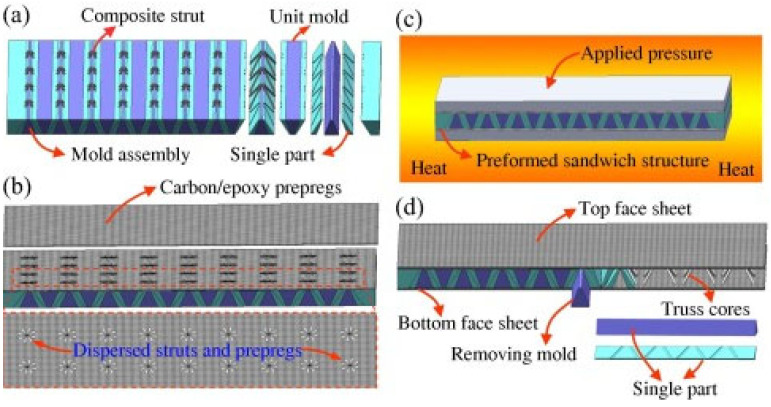
The manufacturing process of sandwich composites: (**a**) assembly of the mold and unit parts; (**b**) placement of prepregs and reinforcements; (**c**) application of pressure and heat to the preformed structure; (**d**) removal of the mold and finalization of the sandwich [40].

As shown in Figure 19, the manufacturing process of sandwich composites begins with assembling the mold and arranging prepregs and reinforcements (parts a, b). Subsequently, pressure and heat are applied to the preformed structure, ensuring strong bonding between layers (part c). The final step involves removing the mold and finishing the sandwich component (part d), which achieves the desired mechanical and thermal properties [38].

The flexibility and mechanical resistance of carbon composites were also analyzed under extreme temperatures and various loading modes. Results showed that temperatures exceeding 2000 °C significantly influence the strength and failure mechanisms of these materials. Under Y-direction loading, linear load–deformation behavior persists, while Z-direction loading results in nonlinear changes due to interlaminar defects. With increasing temperature, strength improves in both directions, with flexural strength in the Y-direction rising by 55.6% and in the Z-direction by 188.5% at 2000 °C. This improvement is accompanied by increased modulus of elasticity, confirming the substantial effect of temperature on the structural properties of composites [30,50].

The failure mechanism of carbon composites at high temperatures, as reported by Chen et al., depends on the loading direction and structural changes. At temperatures above the glass transition point, fiber microwrinkling and stronger interlayer bonding reduce the likelihood of delamination. Z-direction loading also exhibits greater resistance to damage due to enhanced matrix integrity, which helps maintain mechanical properties even under intense thermal loading [50].

These findings demonstrate that the combination of nanostructured reinforcements, an optimized manufacturing process, and high structural integrity enables carbon composites to achieve exceptional mechanical properties under extreme conditions. These results open new possibilities for their applications in the aerospace and space industries, where high strength, thermal resistance, and low weight are critical requirements [30,38,40,50].

### 5.2. Dynamic and High-Temperature Behavior of Composites

Dynamic loading and high temperatures place extreme demands on composite materials, affecting their mechanical resilience, structural integrity, and long-term durability. Carbon–carbon (C/C) composites, C/C-SiC composites, and ceramic matrix composites reinforced with continuous fibers represent advanced solutions for challenging conditions encountered in aerospace, railway transportation, and energy sectors [25,52,53].

**Figure 20:**
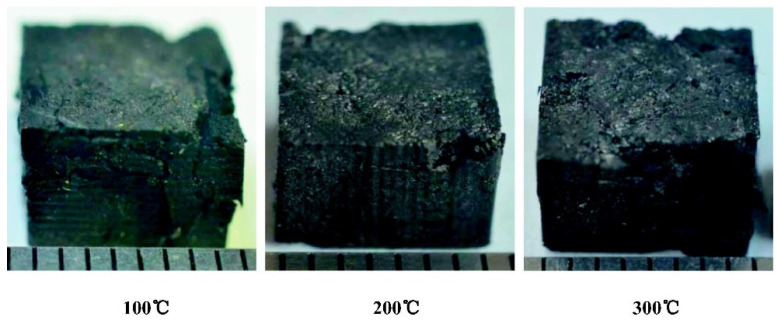
Thermal degradation of composites at various temperatures: 100 °C, 200 °C, and 300 °C [53].

Figure 20 illustrates the thermal degradation of composites at various temperatures (100 °C, 200 °C, and 300 °C), showing progressive structural damage. At lower temperatures, surface integrity is maintained, whereas higher temperatures result in more extensive degradation, emphasizing the importance of optimizing composites for high-temperature applications. These visual differences highlight the necessity for advanced solutions to preserve structural stability under intense thermal and mechanical loads [53].

The mechanical properties of 3D needled C/C composites, as shown in the research by Yu et al., are significantly influenced by strain rate and temperature. At high loading rates, these materials exhibit increased strength and stiffness, while various damage mechanisms occur, including matrix cracking, fiber delamination, and shear fracture. With rising temperatures, both the strength and modulus of elasticity of the composites increase; however, at extreme temperatures above 300 °C, these materials become more brittle, with shear and delamination damage predominating [53].

C/C-SiC composites, as reported by Choudhury et al., used in the brake discs of high-speed trains, exhibit significant changes in mechanical properties at high temperatures. Studies have shown that at temperatures above 600 °C, oxidation and thermal expansion lead to a reduction in compressive, tensile, and flexural strength by 25–41%. Conversely, at lower temperatures up to 200 °C, stress relaxation at the fiber–matrix interface can temporarily enhance the material’s strength [53].

**Figure 21:**
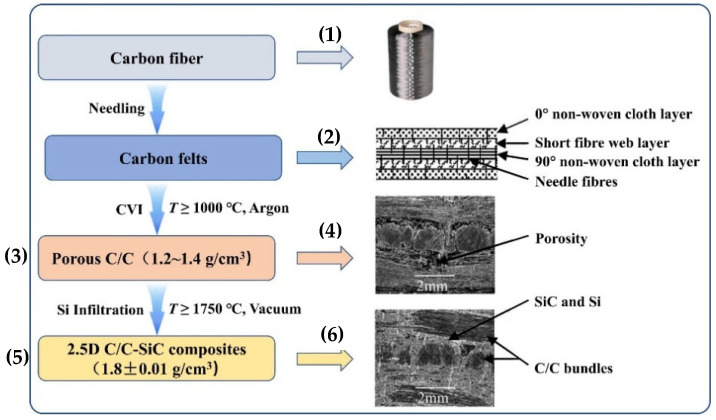
The manufacturing process of 2.5D C/C-SiC composites: (**1**) carbon fibers and needling; (**2**) production of carbon felts; (**3**) infiltration via CVI at T ≥ 1000 °C (argon atmosphere), (**4**) porous C/C composite; (**5**) Si infiltration at T ≥ 1750 °C (vacuum); (**6**) resulting C/C-SiC composite [21].

Figure 21 illustrates the manufacturing process of 2.5D C/C-SiC composites, which involves several steps from needling carbon fibers to Si infiltration at high temperatures. This process achieves the required high mechanical strength and thermal resistance of the material [21].

Continuous fiber-reinforced ceramic matrix composites (CFR CMCs), as highlighted by Li et al., outperform traditional materials with their ability to withstand temperatures up to 1600 °C, reducing the need for cooling. These properties make them suitable for use in the hot sections of aircraft engines, such as combustion chambers, turbine blades, and nozzles. Furthermore, the increased thermal resistance by 200–350 °C enhances the reliability and operational efficiency of engines [25].

These advanced composite materials demonstrate significant improvements in dynamic and high-temperature properties, making them indispensable for applications where mechanical reliability and thermal stability are paramount [21,25,52,53].

## 6. Advanced Materials and Manufacturing Technologies: Development of Advanced Composites for Extreme Conditions

Advanced composites for extreme conditions combine the properties of epoxy resins and ceramic fillers, ensuring high strength and thermal stability. Curing processes, such as controlled temperature profiles, significantly influence their mechanical properties and long-term durability. Biologically derived epoxy resins offer sustainable solutions while maintaining exceptional strength and thermal resistance. These technologies enable the production of sustainable and high-performance composites for modern industrial applications [39,40,54,55].

Advanced composite materials are essential for applications in extreme conditions, such as hypersonic flights, combustion chambers, rocket engines, and other demanding environments. Key properties for these applications include high strength, thermal stability, and resistance to ablation and mechanical damage. Modern research focuses on developing ceramic matrix composites (CMCs) and carbon-fiber-reinforced polymer composites with advanced modifications [54,55].

Epoxy oligomers with epoxy and hydroxyl groups are frequently utilized as binders in the fabrication of composites with enhanced mechanical and physical properties, such as adhesion, cohesion, impact strength, and thermal resistance. These properties are further improved by reinforcing the epoxy binder with dispersed mineral particles, where the physicochemical processes at the interface between the binder and the filler play a crucial role in forming the composite structure. The outer surface layers are formed through physical and chemical interactions between the particle surfaces and macromolecules, enhancing both adhesion and cohesion of the material and significantly influencing the mechanisms of deformation and fracture [56].

Epoxy composites exhibit a high degree of heterogeneity, which is a critical factor in their physico-mechanical properties. During dynamic deformation, a hierarchy of structural scales is formed, where microscopic cracks initiate macroscopic processes leading to material failure. A composite with SiC filler (40%) achieves the highest fracture toughness, making it suitable for applications involving high-impact loads, although a higher filler content results in increased brittleness of the material. Increasing the volumetric fraction of the filler leads to the formation of surface layers that enhance the overall properties of the composites, demonstrating potential for further improvements in their operational characteristics [56].

Ultra-high-temperature ceramic composites (UHTCs), such as ZrB_2_ and HfB_2_, have proven effective in applications where temperatures exceed 3000 °C. These materials combine extreme thermal stability with ablation resistance, making them suitable for aerodynamic leading edges and nozzles of hypersonic vehicles. However, their brittleness remains a challenge addressed by reinforcement with 3D carbon fibers. This structure enhances material toughness, while pressure infiltration of solid and liquid phases improves homogeneity and mechanical stability. The resulting composites exhibit flexural strength of up to 603 MPa and exceptionally low ablation rates at 2500 °C [54].

**Figure 22:**
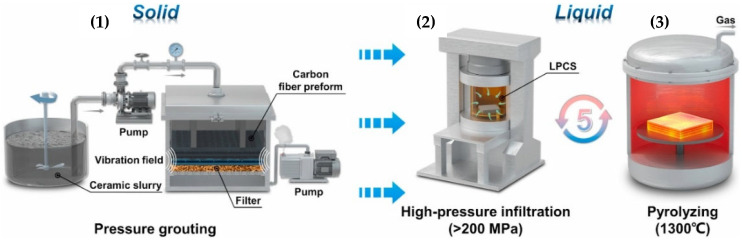
Composite manufacturing process: (**1**) pressure injection of ceramic slurry into a carbon preform; (**2**) high-pressure infiltration (>200 MPa), (**3**) pyrolysis at 1300 °C [36].

Figure 22 illustrates the process of manufacturing ceramic composites, which involves pressure injection of ceramic slurry into a carbon preform, subsequent high-pressure infiltration (>200 MPa), and pyrolysis at 1300 °C. This process is essential for achieving the mechanical properties and thermal resistance required for composites designed for extreme conditions [36].

Carbon-fiber-reinforced polymers (CFRPs) represent another significant direction in the development of composites for extreme conditions. The introduction of aramid nanofibers (ANFs) brings notable improvements in interlaminar strength, toughness, and thermal stability. ANFs bond to the epoxy matrix through hydrogen bonding and π–π interactions, resulting in up to a 5.1-fold increase in tensile strength and enhanced short beam strength at high temperatures. The thermal stability of these hybrid composites improved by over 30 °C, and their resistance to thermal decomposition was 33.7% higher compared to conventional CFRPs [55].

**Figure 23:**
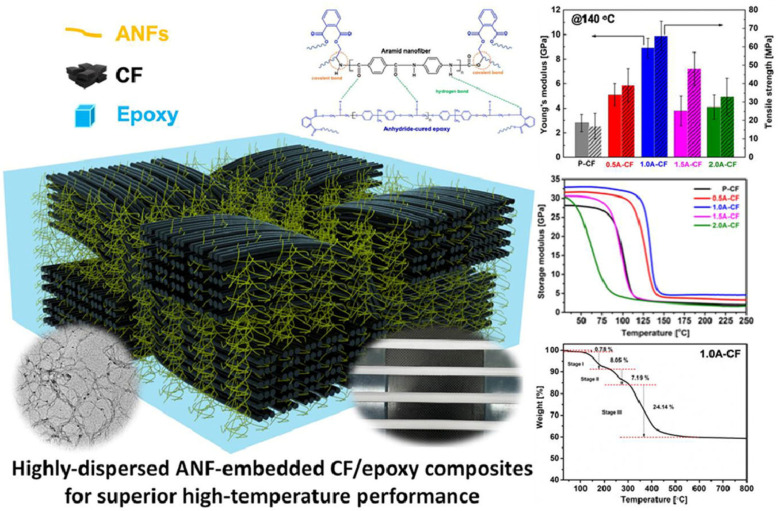
CF/epoxy composites with ANFs: highly dispersed aramid nanofibers (ANFs) for exceptional performance at elevated temperatures—structure, thermal resistance, and mechanical properties [57].

Figure 23 depicts CF/epoxy composites with ANFs, where highly dispersed aramid nanofibers enhance the material’s performance at elevated temperatures. The figure illustrates the structure, thermal resistance, and mechanical properties of these composites, which exhibit exceptional performance under demanding conditions [36,55].

Fiber–metal laminates (FMLs) combine metal sheets with fiber-reinforced polymers, achieving excellent resistance to fatigue stress and impact. In aerospace applications, FMLs like ARALL and GLARE, which combine aluminum alloys with aramid or glass fibers reinforced with epoxy, are widely used. In efforts to reduce weight, magnesium alloys are increasingly explored as substitutes for aluminum sheets. This approach reduces laminate density by approximately 20% but also leads to a decrease in their static properties. To enhance the mechanical properties of magnesium-based FMLs, the use of carbon-fiber-reinforced polymer (CFRP) is being considered [13].

This research focused on the influence of surface roughness of magnesium sheets on interlaminar strength, showing that rougher surfaces improve peel and shear strength compared to smoother surfaces. These findings confirm the benefits of sandblasting as a method to improve adhesion [13].

The study of advanced composites underscores the importance of innovations in material manufacturing for extreme conditions. The combination of ultra-high-temperature ceramics and modified CFRP materials opens new possibilities in aerospace, space exploration, and other industries where the demands for strength, stability, and durability are at the highest level [13,54,55,57].

### 6.1. Curing Processes and Their Impact on Mechanical Properties

Epoxy resins are one of the most crucial components of advanced composites, widely used in industry due to their high strength, chemical resistance, and ability to withstand mechanical loads. The curing process of epoxy resins has a significant impact on the final mechanical properties of composites, making its optimization essential for improving material performance [39,40].

The curing of epoxy resins involves an exothermic reaction that forms a three-dimensional polymer network. This process is influenced by temperature, curing time, and the hardeners used. Proper control of the curing process is essential to ensure uniform reaction progress and prevent defects such as incomplete conversion of epoxy groups or internal stresses. One of the most effective methods for monitoring this process is FTIR spectroscopy, which tracks the concentration of reactive groups during curing and analyzes the reaction mechanisms [40]. Studies by Yao et al. have shown that optimal curing conditions directly affect the strength and toughness of composites. For example, curing the Aerotuf 275-34TM epoxy resin at 160 °C for 15 min achieved a conversion degree of 0.91, representing an optimal balance between mechanical properties and process efficiency. Carbon-fiber fabric laminates oriented at [±45°] angles achieved the best tensile strength under these conditions [39].

Dynamic mechanical analysis (DMA) provides additional insights into the viscoelastic properties of epoxy composites during curing. Testing the storage modulus (E′) and loss modulus (E″) reveals the material’s ability to store and dissipate energy. Adding fillers such as graphite to the epoxy matrix significantly improves the mechanical and thermal properties of the composites. The storage modulus of epoxy resins with graphite filler was higher both above and below the glass transition temperature (Tg), indicating greater stiffness and resistance to deformation. These results underscore the importance of fillers not only for enhancing strength but also for optimizing the curing process [40].

In addition to curing parameters, the choice of resin type and modification method also plays a critical role. Adding thermoplastic components, such as polysulfone (PSF), can improve the toughness of epoxy resins without negatively affecting their compatibility with carbon fibers. Modifying the epoxy matrix with PSF increases thermal stability and reduces brittleness, resulting in better mechanical properties of composites under various conditions [39].

Together, these factors demonstrate that proper adjustment of the curing process and the appropriate selection of fillers or additives can significantly enhance the performance of epoxy composites. Optimization of the process not only increases the mechanical resilience of materials but also extends their service life and broadens their application in industries such as aerospace, automotives, and construction [39,40].

### 6.2. Biologically Derived Epoxy Resins

Epoxy resins are an integral part of advanced composite materials, but their traditional petroleum-based formulations, such as diglycidyl ether of bisphenol A (DGEBA), pose environmental challenges. The production of these resins involves chemicals that can negatively impact health and the environment, including carcinogenic effects and endocrine disruption. As a result, there is growing interest in the development of biologically derived epoxy resins as more eco-friendly and sustainable alternatives.

Biologically derived compounds, such as lignin, vanillin, eugenol, and their derivatives, provide a promising foundation for the development of sustainable epoxy resins. These compounds contain phenolic rings, which are ideal for creating high-performance polymers. Recent studies have shown that lignin-based resins, such as spiro diacetal epoxy resin (SDE), exhibit mechanical properties comparable to traditional DGEBA epoxy resins while offering the advantages of biodegradability and lower environmental impact [41].

A key challenge in using biologically derived epoxy resins is ensuring sufficient interfacial adhesion between carbon fibers and the resin matrix. The surface of carbon fibers is chemically inert and smooth, which can result in weak interactions with the matrix. Techniques such as plasma treatment, surface modifications, or the application of sizing agents are employed to address this issue. Plasma treatment can increase the surface roughness of fibers and introduce chemically active groups, improving stress transfer between the fibers and the matrix.

**Figure 24:**
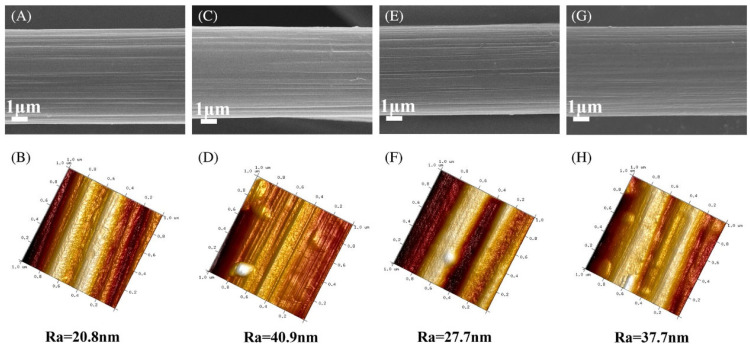
Surface morphology and roughness of carbon fibers: (**A**,**C**,**E**,**G**) SEM images, (**B**,**D**,**F**,**H**) AFM analysis with Ra (roughness) values [41].

Figure 24 illustrates the surface morphology and roughness of carbon fibers, with SEM images of the surface shown in panels (A, C, E, G) and AFM analysis with Ra (roughness) values in panels (B, D, F, H). These images highlight differences in fiber surface structure and roughness, which can significantly influence the quality of interfacial adhesion in composites [41].

New studies, as reported by Xu et al., have shown that biologically derived epoxy emulsions can significantly improve the mechanical properties of composites. The emulsion prepared from spiro diacetal epoxy resin (S-P-S) increases the oxygen content on the surface of carbon fibers and creates a gradient interfacial layer with a modulus gradually changing between the fibers and the matrix. This layer allows for more efficient stress transfer and enhances the strength of the composites. Compared to the commercial DGEBA-based epoxy emulsion, S-P-S demonstrated a significant improvement in interfacial properties, including shear strength and fiber transverse strength [35].

In addition to ecological advantages, biologically derived epoxy resins also offer excellent thermal stability and mechanical performance. Testing of biologically derived resins in composites showed increased toughness, improved crack resistance, and lower moisture absorption compared to traditional epoxy resins. These properties make biologically derived resins attractive for a wide range of industrial applications, including the automotive, aerospace, and sports industries. The development of biologically derived epoxy resins represents a significant step towards sustainability in the field of composite materials. With a combination of ecological benefits and excellent mechanical properties, these resins can replace traditional materials in various industrial sectors, contributing to reducing environmental impact and supporting green technologies [29,41].

## 7. Microstructure and Nondestructive Analysis of Composites: Porosity and Microstructure in C/C Composites

The control of the microstructure of composites, including porosity and fiber distribution, is crucial for ensuring their strength and resistance to loading. Optimizing the bonding between the fibers and the matrix reduces defect formation and improves mechanical properties. Nondestructive methods, such as infrared thermography and experimental modal analysis, allow for the identification of damage such as delaminations and microcracks without the need for disassembling the material, thereby extending the lifespan and increasing the reliability of composites [58].

**Figure 25:**
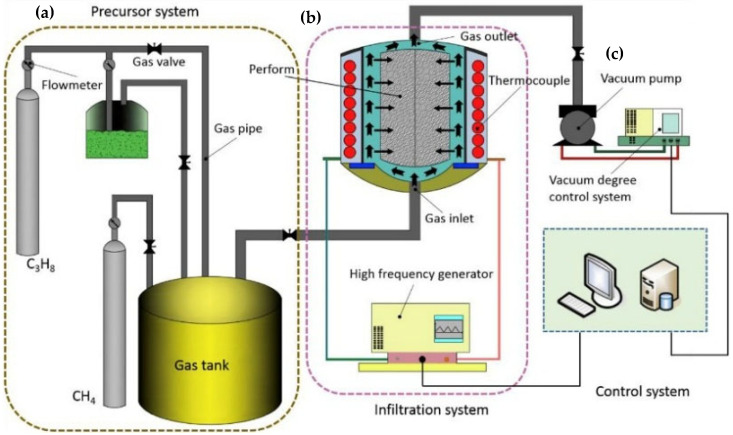
Gas Infiltration System Schematic: (**a**) precursor system (C_3_H_8_, CH_4_); (**b**) infiltration system with high-frequency generator; (**c**) vacuum system with pressure and temperature control [59].

Porosity and microstructure are key factors influencing the mechanical properties of carbon–carbon (C/C) composites, which are widely used in the aerospace and space industries. Chemical vapor infiltration (CVI), one of the main manufacturing methods, leads to the formation of pores in the material that significantly affect the strength of the composites. Increased porosity causes stress concentration, leading to a dramatic decrease in mechanical properties—for example, the flexural strength can decrease by up to 57% when porosity reaches 18%. The main damage mechanisms are delamination and fiber damage in critical layers. Figure 25 shows a schematic of the gas infiltration system, which includes a precursor system (C_3_H_8_, CH_4_), an infiltration system with a high-frequency generator, and a vacuum system with pressure and temperature control. This process is critical for improving matrix distribution and controlling porosity during composite manufacturing, thus enhancing their mechanical properties and resistance [33,58].

The selection of suitable carbon fibers plays a crucial role in controlling porosity and improving the properties of the material. T300 carbon fibers, with a specially designed rough surface structure, allow for a stronger bond between the fibers and the pyrolytic carbon matrix, minimizing pore size and enhancing strength. In contrast, TZ300 carbon fibers, while achieving higher graphitization, are less resistant to mechanical stress after high-temperature processing. This property makes them less suitable for applications requiring high structural integrity [58].

The use of needle-punching technology (2.5D preform), as described in the research by Deng et al., improves the interlayer bonding, reducing the risk of delamination. This process also enables a higher matrix density and better mechanical stability of the overall composite. Numerical simulations and experimental measurements further emphasize the importance of optimizing the microstructure to minimize defect formation and maximize the mechanical properties of C/C composites [60].

These findings underscore the importance of controlling porosity and optimizing the microstructure for the development of advanced carbon composites that meet demanding requirements under high temperatures and loads, such as in rocket engines and aerodynamic brakes [58,60].

### Nondestructive Damage Detection in Composites

Reliable damage detection in composites is crucial for their safe and efficient use in various industrial applications, including aerospace, railway transportation, and the energy industry. Modern nondestructive methods, such as laser infrared thermography (LIT) and experimental modal analysis (EMA), provide accurate tools for identifying and characterizing damage without the need for disassembling the material. These methods are essential for the long-term monitoring and maintenance of composite structures [33].

**Figure 26:**
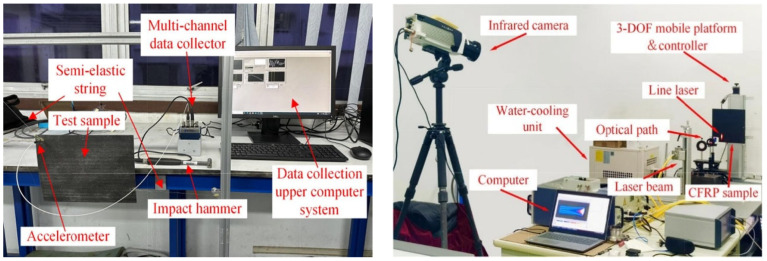
Experimental Setup for Dynamic Testing: Sample on Semi-Rigid String, Accelerometer, Impact Hammer, Multi-Channel Data Collector, and Data-Processing System [33].

Figure 26 shows the experimental setup for dynamic testing of composite materials. On the left side, the setup includes a sample on a semi-rigid string, accelerometer, impact hammer, multi-channel data collector, and data-processing system. On the right side is the infrared thermography setup, utilizing a linear laser and optical path for detailed monitoring of damage in carbon-fiber-reinforced polymer (CFRP) samples [33].

**Figure 27:**
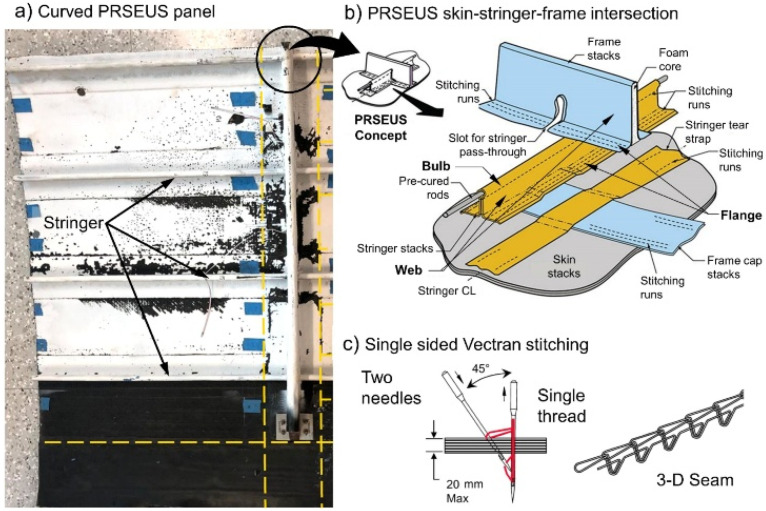
Inner mold line (IML) view of: (**a**) a curved PRSEUS panel (not to scale); (**b**) exploded schematic of PRSEUS skin–stringer–frame intersection, adapted from; (**c**) single-sided stitching forming a 3D seam chain-stitch, adapted from [52].

LIT is a highly effective technology for detecting surface and subsurface defects, utilizing temperature differences stimulated by thermal waves. EMA complements LIT by analyzing the dynamic properties of materials, such as natural frequencies and mode shapes, revealing the effect of defects on structural integrity. The combination of these methods allows for reliable prediction of the size and depth of defects, with bivariate models like LE-SVM achieving high detection accuracy. Specific setups for experimental verification of these detection methods are often used to thoroughly analyze the mechanical and dynamic properties of composites. Figure 27 shows the experimental setup for dynamic testing, which includes a sample on a semi-rigid string, accelerometer, impact hammer, and multi-channel data collector. This approach is effective in assessing the composite’s response to dynamic loading and provides detailed data that are essential for analyzing damage and defects that may impact the material’s structural integrity [33].

The topic of reliable damage detection in composites directly aligns with research focused on predicting the properties of polymer composites using machine learning methods. Polymer composite materials are widely utilized in the aerospace industry for the production of load-bearing structures, such as fuselages, wings, and interior components, due to their excellent physical–mechanical and thermophysical properties. Their development necessitates the optimization of fillers, filler content, and polymerization conditions to achieve superior technological and operational characteristics.

Advanced machine learning methods, particularly neural networks and boosted decision trees, enable highly accurate predictions of composite properties, such as thermal conductivity, fatigue life, and stress–strain characteristics. Studies have demonstrated that these methods can simulate the thermal conductivity of composites reinforced with various fibers (glass, carbon) and filled with materials such as aerosil, γ-aminopropyl aerosil, aluminum oxide, and chromium oxide, with prediction errors ranging from only 0.2% to 1.5% [51].

Understanding thermal conductivity is crucial for designing materials with optimized properties. Such materials find applications not only in aerospace but also in construction and electronics, improving the efficiency, reliability, and durability of structures. Machine learning thus opens new possibilities for the development of modern composites with precisely defined properties [51].

Fractographic analysis provides crucial information about the damage mechanisms of composites, especially in cases where the materials are subjected to combined mechanical and thermal loading. When examining carbon–epoxy PRSEUS structures, it was found that intense exposure to flames causes degradation of the matrix, the formation of porous carbon char, and fiber oxidation. The polyester components in the material dissolve and re-solidify, which can mask primary signs of mechanical failure. In such cases, Vectran™ stitches are much more resistant to thermal damage compared to conventional polyester materials [61].

Research by Chao et al. on the effect of temperature on the mechanical properties of carbon-fiber-reinforced polymers (CFRPs) shows that these materials are sensitive to extreme temperatures. At low temperatures (−100 °C), they are brittle and prone to damage due to cracks in the matrix, while at high temperatures (100 °C), they soften, leading to fiber detachment from the matrix and ductile failure. A study using micro-CT and digital volume correlation revealed that the shear modulus of CFRP decreased by 78.4% between −100 °C and 100 °C, with increased deformation before failure at higher temperatures. These findings are significant for designing lightweight and durable structures in extreme conditions, such as in aviation [59].

Defects in the form of fiber waviness in laminated composites significantly affect their compressive strength. This study combined experimental testing with numerical simulations and revealed that low-intensity waviness primarily causes fiber failure under compression, while high-intensity waviness leads to delamination between layers. Numerical models incorporating 3D failure criteria accurately predicted the load force levels with a precision of 10% and correctly identified the dominant failure modes for various waviness intensities. Additionally, they uncovered a threshold waviness configuration beyond which the dominant failure mechanism changes. These results help in designing resilient composite structures with tolerance to defects, which is crucial for their industrial application [62].

**Figure 28:**
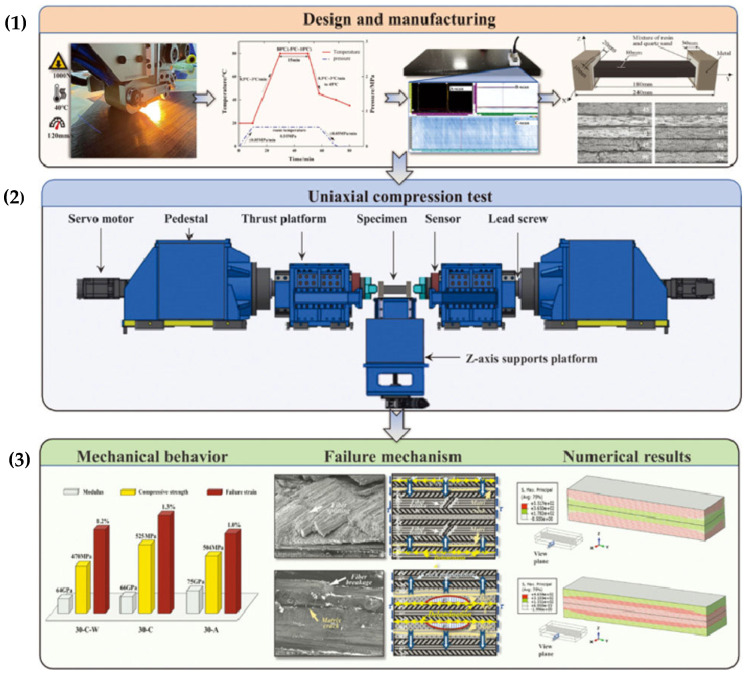
Complex composite analysis: (**1**) design and manufacturing; (**2**) uniaxial compression test with testing equipment; (**3**) mechanical behavior, failure mechanism, and numerical results [63].

To experimentally verify these theoretical models, advanced analytical approaches are used, as illustrated in Figure 28. This process includes (1) material design and manufacturing, where various parameters such as temperature, time, and material components are considered, (2) uniaxial compressive testing with specified testing equipment to measure the mechanical properties of materials, and (3) analysis of mechanical behavior, failure mechanisms, and numerical results, which provide a detailed insight into the behavior of composites under load and assist in optimizing their structural properties [62].

Ultra-thick CFRP laminates are promising materials for deep-sea applications; however, their compressive strength is significantly affected by delamination and fiber wrinkling. A study by Oushab et al. developed a new uniaxial testing method with high accuracy and found that fiber wrinkling reduces compressive strength by 11%, while delamination dominates laminate failure. Axially oriented laminates have lower compressive strength than circumferentially oriented samples, with failure starting from the outer layers and propagating inward. Numerical simulations accurately predicted laminate strength, with predictions based on the Puck failure criterion being most accurate for circumferential samples (0.07% deviation), and the Tsai–Wu 3D criterion for axial samples (5.1% deviation). These findings provide a theoretical basis for the design of ultra-thick laminates, and future research should focus on suppressing delamination and enhancing interlaminar strength in ultra-thick structures [64].

**Figure 29:**
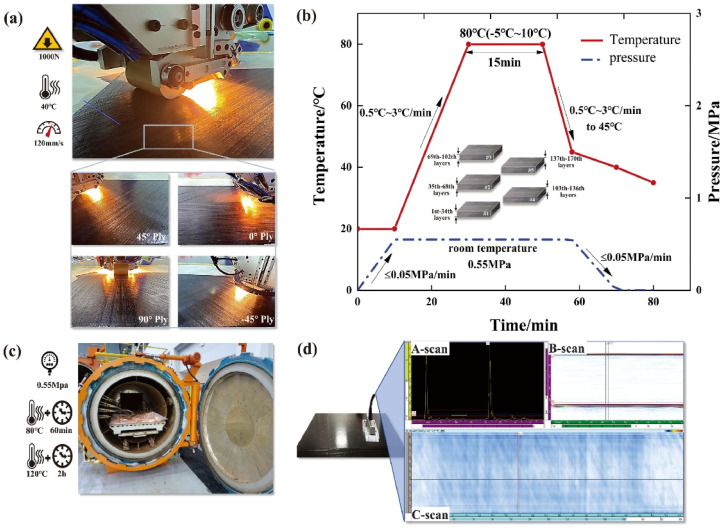
The process of manufacturing and analyzing composites: (**a**) fabrication of layers with different orientations (0°, 45°, 90°); (**b**) temperature and pressure profile during curing; (**c**) autoclave curing of composites; (**d**) ultrasonic testing (A-scan, B-scan, C-scan) [64].

Figure 29 illustrates the entire process of manufacturing and analyzing composites, which includes (a) fabricating layers with different fiber orientations (0°, 45°, 90°), (b) the temperature and pressure profile during curing, (c) autoclave curing of composites, and (d) ultrasonic testing (A-scan, B-scan, C-scan). This comprehensive approach allows for detailed monitoring of the manufacturing process while evaluating material quality during testing, which is essential for optimizing their performance and reliability [64]. 

Fiber-reinforced composite materials are increasingly used in industry due to their exceptional properties, but they may contain defects that reduce their performance. This study highlights the importance of infrared (IR) thermography in nondestructive testing of composites. The results showed that defect detection, such as delaminations, depends on the type of reinforcement, fiber configuration, and defect depth. The best results were achieved with carbon composites, where defects were detected up to a depth of 2.3 mm. Optimal conditions for detection include a longer heating time, monitoring the sample’s cooling, and adjusting the detection time according to the defect’s depth. These insights are crucial for evaluating the technical condition of composites during manufacturing and operation, thereby enhancing their reliability and safety [63].

These findings emphasize the importance of combining nondestructive methods with fractographic analysis for comprehensive damage diagnostics in composites. The integration of innovative technologies with traditional analytical methods not only extends the lifespan of composite materials but also increases their safety in demanding applications [33,60,61,62,63,64].

## 8. Future Developments

Epoxy composites have the potential for further development in terms of their properties and applications, which could significantly impact their use in a wide range of industries. The prospects for future development are based on improving mechanical properties, standardizing for extreme conditions, and developing sustainable materials.

• **Improvement of mechanical properties:** • Increase tensile strength to values above 120 MPa, allowing epoxy composites to be used in even more demanding structures such as bridge structures and highly loaded aerospace components. • Increase the modulus of elasticity above 12 GPa, making the materials even more suitable for applications with extreme stiffness requirements, such as space research. • Development of composites with resistance to multi-axial cyclic loads, significantly improving their service life in robotics and automotive components. **Applications:** These improvements are suitable for use in infrastructure such as bridges, aerospace components like airplane fuselages and wings, space research structures, robotics, and automotive suspension systems.

• **Resistance to extreme temperatures:** • Ensure stable mechanical properties over a wider temperature range from −50 °C to 200 °C, enabling their use in even harsher conditions. • Development of special epoxies based on ceramic additives that can maintain strength parameters even at 300 °C, opening up opportunities for applications in nuclear energy. • Improvement of temperature resistance for epoxies intended for freezing and cryogenic applications to minimize brittleness at temperatures close to absolute zero.

**Applications:** These advancements are applicable in aerospace, automotive components, high-temperature industrial systems, nuclear energy applications, cryogenic storage systems, and space exploration technologies.

• **Optimization of the curing process:** • Controlled curing using nanoadditives that improve material homogeneity while reducing internal stress by 20%. • Introduction of adaptive curing methods that allow precise adjustment of material properties according to the requirements of specific applications. • Application of advanced technologies, such as laser curing, to minimize production time without compromising quality and precision.

**Applications:** These improvements are applicable in precision automotive parts, aerospace components, medical devices, high-performance sports equipment, electronics manufacturing, and other sectors requiring fast, high-quality production methods.

• **Development of environmentally sustainable epoxies:** • Increase the share of biologically derived epoxies in the market to at least 40% by 2035. • Development of biological epoxies resistant to corrosive chemicals and UV radiation for outdoor applications. • Improvement of the properties of ecological epoxies, such as increased resistance to moisture and temperature changes, to a level comparable to petrochemical variants. **Applications:** These advancements are applicable in sustainable construction, green technology, automotive parts, outdoor coatings, marine applications, renewable energy systems, and other environmentally conscious industries.

• **Hybrid and nanocomposites:** • Combination of graphene with carbon nanotubes to achieve synergistic effects that improve the electrical and mechanical properties of materials by up to 30%. • Integration of ceramic nanoparticles to improve thermal conductivity to values above 8 W/mK, enabling their use in high-performance electronics. • Development of multi-layered hybrid structures combining epoxies and elastomers, enhancing their resistance to impact and cracking. **Applications:** These innovations are applicable in lightweight vehicles, energy storage systems, advanced structural components, high-performance electronics, heat sinks, automotive panels, aerospace safety components, and impact-resistant materials.

In the following Table 1, various additives and processes used in the production of epoxy composites are summarized, along with their impact on mechanical, thermal, tribological, and ecological properties. Each type of additive or process provides specific benefits that can be utilized in various applications.

## 9. Conclusions

Epoxy polymers are the foundation of modern composite materials, which find applications in industries such as aerospace, automotives, construction, and energy. Their exceptional properties, such as high strength, thermal stability, and resistance to chemical influences, allow the creation of reliable and effective solutions for a wide range of applications.

The mechanical properties of epoxy composites, such as tensile strength, modulus of elasticity, and crack resistance, have been significantly improved by the use of nanomaterials, fibrous reinforcements, and innovative manufacturing technologies. Research in the field of epoxies has highlighted the following key strategies for their optimization:

- **Improvement of thermal stability:** The addition of ceramic fillers, such as titanium dioxide or silica, significantly increases the resistance of epoxies to high temperatures and thermal degradation.
- **Optimization of mechanical properties:** The combination of fibrous fillers (carbon fibers) and particulate fillers (nanoparticles) enhances tensile strength, modulus of elasticity, and resistance to delamination.
- **Innovative surface treatments:** Coatings based on polydopamine or hybrid chemical modifications significantly improve the adhesion between the matrix and reinforcement, enabling more efficient stress transfer.
- **Thermal and environmental resistance:** The use of bio-based resins and ecological curing agents minimizes the environmental impact without compromising thermal resistance.
- **Hybridization and multi-functional properties:** The combination of epoxy matrices with nanotechnologies (oxidized graphene, carbon nanotubes) creates materials with unique properties, such as electrical conductivity or self-healing capabilities.

These innovations not only bring significant improvements to the properties of epoxy composites but also expand their use in environments with extreme demands for strength and thermal resistance. In this way, epoxy composites will gradually adapt to the needs of modern industry while contributing to improving environmental sustainability.

## Figures and Tables

**Table 1:** Comparison of additives, processes, and their properties.

Additives/Processes	Mechanical Properties	Thermal Properties	Tribological Properties	Ecological Properties
Nanomaterials: Graphene	Improved conductivity, tensile strength, thermal shock resistance	Higher stability, aging resistance	None	None
Nanomaterials: TiO_2_	Higher tensile strength, fatigue resistance	Improved thermal stability and resistance to high temperatures	None	None
Nanomaterials: SiO_2_	Better interlayer strength, stiffness, delamination resistance	None	None	None
Nanomaterials: Graphite	Improved wear resistance, compressive strength	Better thermal shock resistance	Reduced friction, extended lifespan	None
Nanomaterials: CNTs	Enhanced stress transfer, interlayer strength	Improved shock resistance	None	None
Hybrid reinforcement: Carbon + Aramid fibers	Better adhesion, reduced microcracks	None	None	None
Hybrid reinforcement: CNTs + Graphene	Synergistic effects, improved properties	Thermal and mechanical load resistance	None	None
Bio-based resins: Lignin	Sustainable, strong	Sustainable, thermally stable	None	Sustainable, strong
Bio-based resins: Vanillin	Moisture and UV resistance, eco-friendly	None	None	Eco-friendly, UV/moisture resistant
Tribological additives: PTFE + PES/PMPS	None	None	Lower friction, better wear resistance	None
Ceramic fillers: SiC	Higher compressive strength, thermal cycle resistance	High stability, wear resistance	Improved wear resistance, compressive strength	Lower CO_2_ emissions, eco-alternative
Ceramic fillers: Al_2_O_3_	Better properties at high temperatures	High stability, wear resistance	None	None
Ceramic fillers: TiO_2_	None	Higher thermal stability	None	None

## Data Availability

Not applicable.
